# Divergent *Hemogen* genes of teleosts and mammals share conserved roles in erythropoiesis: analysis using transgenic and mutant zebrafish

**DOI:** 10.1242/bio.035576

**Published:** 2018-08-10

**Authors:** Michael J. Peters, Sandra K. Parker, Jeffrey Grim, Corey A. H. Allard, Jonah Levin, H. William Detrich

**Affiliations:** Department of Marine and Environmental Sciences, Northeastern University, Nahant, MA 01908, USA

**Keywords:** Anemia, CRISPR/Cas9, Gene editing, Hematopoiesis, Transcription

## Abstract

Hemogen is a vertebrate transcription factor that performs important functions in erythropoiesis and testicular development and may contribute to neoplasia. Here we identify zebrafish Hemogen and show that it is considerably smaller (∼22 kDa) than its human ortholog (∼55 kDa), a striking difference that is explained by an underlying modular structure. We demonstrate that Hemogens are largely composed of 21-25 amino acid repeats, some of which may function as transactivation domains (TADs). *Hemogen* expression in embryonic and adult zebrafish is detected in hematopoietic, renal, neural and gonadal tissues. Using *Tol2-* and CRISPR/Cas9-generated transgenic zebrafish, we show that *Hemogen* expression is controlled by two Gata1-dependent regulatory sequences that act alone and together to control spatial and temporal expression during development. Partial depletion of Hemogen in embryos by morpholino knockdown reduces the number of erythrocytes in circulation. CRISPR/Cas9-generated zebrafish lines containing either a frameshift mutation or an in-frame deletion in a putative, C-terminal TAD display anemia and embryonic tail defects. This work expands our understanding of Hemogen and provides mutant zebrafish lines for future study of the mechanism of this important transcription factor.

## Abstract (precis)

**Summary:** Transgenic and mutant zebrafish lines were created to characterize the expression and functions of Hemogen, a transcription factor involved in the formation of red blood cells and other processes.

## INTRODUCTION

Hemogen (Hemgn) is a vertebrate transcription factor that is expressed in mammalian hematopoietic progenitors (Lu et al., 2001; Yang et al., 2001) and has been implicated in erythroid differentiation and survival (Li et al., 2004). Originally identified in mice and subsequently described in humans as EDAG (Erythrocyte Differentiation Associated Gene), Hemogen has also been implicated in testis development in mammals and chickens (Nakata et al., 2013; Yang et al., 2003), and in osteogenesis in rats (Krüger et al., 2002, 2005). Here we analyze the developmental roles of teleost Hemogen using the zebrafish model system and its powerful suite of reverse-genetic technologies.

Teleost *Hemogen* was discovered using a subtractive hybridization screen designed to isolate novel erythropoietic genes from fish belonging to the largely Antarctic suborder Notothenioidei (Detrich and Yergeau, 2004; Yergeau et al., 2005). Sixteen species belonging to the icefish family (Channichthyidae) are unique among vertebrates because they are white-blooded; they fail to execute the erythroid genetic program or produce hemoglobin (Cocca et al., 1995; Near et al., 2006; Zhao et al., 1998). Forty-five candidate erythropoietic cDNAs were recovered using representational difference analysis (Hubank and Schatz, 1999) applied to kidney marrow transcriptomes of two notothenioid species, one red-blooded and the other white-blooded (Detrich and Yergeau, 2004; Yergeau et al., 2005). One of the unknown genes, clone *Rda130*, was similar to mammalian *Hemogen* and was expressed only by the red-blooded notothenioid.

Although Hemogen is clearly involved in hematopoiesis, its mechanism remains incompletely understood. In human cell lines, Hemogen activates erythroid gene transcription in part by recruiting the histone acetyltransferase P300 to acetylate Gata1 (Zheng et al., 2014). Like Gata1, Hemogen protects erythroid cells from apoptosis by upregulating anti-apoptotic factors (e.g. Nf-κB*,* Bcl-xL) that are critical for terminal differentiation (Li et al., 2004; Rhodes et al., 2005; Zhang et al., 2012).

The regulation of *Hemogen* expression is of interest because it is overexpressed frequently in patients with a variety of cancers and leukemias (An et al., 2005; Forbes et al., 2017; Li et al., 2004). This putative oncogene, which is located in a human chromosomal region (9q22) of leukemia-associated breakpoints, has been linked to proliferation and survival of leukemic cells and to induction of tumor formation in mice (Chen et al., 2016; Lu et al., 2002). Thus, somatic mutations in *Hemogen* or its regulators may contribute to neoplasia.

The zebrafish is a well-established model organism for studying hematopoiesis in vertebrates because it produces the same blood lineages as mammals (de Jong and Zon, 2005; Paffett-Lugassy and Zon, 2005). In zebrafish, erythropoiesis occurs in sequential waves at unique anatomical locations in embryos and adults that correspond to analogous sites in mammals (Galloway and Zon, 2003). Many of the molecular players that orchestrate the erythroid program appear to be conserved between zebrafish and mammals, but relatively few have been functionally characterized in zebrafish. Nevertheless, mutant zebrafish models accurately phenocopy human blood diseases caused by mutations in major erythroid factors, such as *Gata1* (Lyons et al., 2002) and *Erythroid beta-spectrin* (Liao et al., 2000).

The purpose of this study is to characterize the regulation of *Hemogen* expression and the function of the Hemogen protein in zebrafish. We identify the zebrafish *Hemogen* ortholog, which despite being only 40% as large as the human protein, contains similarly arranged functional motifs. *Hemogen* is expressed in blood, testis, ovaries, kidneys and the central nervous system in zebrafish. Two tissue-specific, alternative *Hemogen* promoters are associated with conserved noncoding elements (CNEs) and have distinct regulatory functions in primitive and definitive hematopoiesis and other processes. By analysis of morphant and mutant zebrafish, we show that Hemogen is required for normal erythropoiesis and that this role depends in part on a cluster of acidic residues within a putative, C-terminal transactivation domain (TAD).

## RESULTS

### Teleosts contain a single *Hemogen*-like gene that is syntenic with human Hemogen

**Fig. 1.**
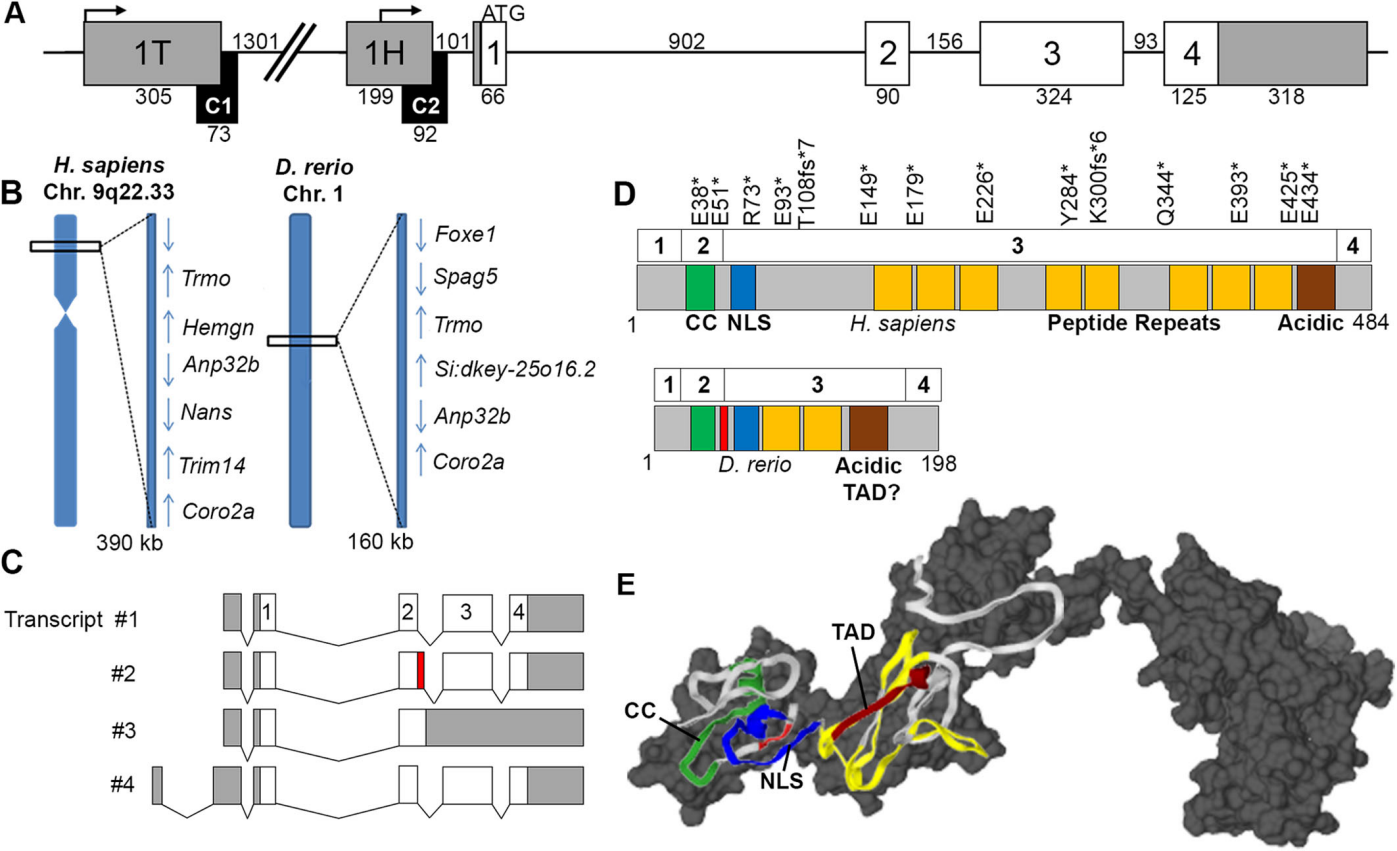
**Zebrafish *Si:dkey-25o16.2* and human *Hemogen* are orthologous and encode related proteins that differ in size.** (A) Structure of the zebrafish *Hemogen*-like gene, *Si:dkey-25o16.2*. Two conserved noncoding elements (C1 and C2, black boxes) were identified in a 2 kb segment proximal to the start codon (see Results, Figs 4-6). Coding exons, white boxes; noncoding exons, gray boxes. Numbers indicate length in bp. (B) Synteny of loci for zebrafish *Si:dkey-25o16.2* on chromosome 1 and *Hemogen* on human chromosome 9 (region q22). Transcriptional orientations indicated by arrows. (C) Alternative splicing of zebrafish *Hemogen*-like transcripts showing sequenced regions. Introns are shown as chevrons. Transcripts 1 and 2 differ by retention of 12 bp of intron (red). (D) Modular structures of zebrafish and human Hemogen proteins each encoded by four exons (numbered boxes). Locations of truncating mutations found in some human cancers (Forbes et al., 2017) are indicated by asterisks. Predicted regions and motifs: green, coiled coil; blue, nuclear localization signal; red, four residues introduced by alternative splicing; yellow, tandem peptide repeats; brown, acidic repeat with transactivation domain (TAD) motif; gray, no prediction. (E) Three-dimensional *ab initio* models of Hemogens. The ribbon diagram of the zebrafish protein, color-coded as in panel D, is superimposed on the gray, space-filling model for the human protein (See Materials and Methods).

Chromosomal synteny is an important criterion when assigning gene relationships across divergent taxa. Despite the whole-genome duplication (WGD) that coincided with the separation of teleosts from more basal ray-finned fishes and tetrapods (Postlethwait et al., 2000), the sequenced genomes of nearly all fishes retain a single *Hemogen*-like gene. We cloned zebrafish *Hemogen*-like cDNAs and found that they corresponded to the predicted gene *Si:dkey-25o16.2* on chromosome 1 of the zebrafish genome (Howe et al., 2013). When we compared the synteny of the putative teleost and mammalian orthologs, represented in Fig. 1B by zebrafish *Si:dkey-25o16.2* (chromosome Dr1) and human *Hemogen* (chromosome Hs9), we found that the flanking genes and their transcriptional orientations were conserved, which strongly supported *Si:dkey-25o16.2* as the zebrafish *Hemogen* ortholog.

### Structure of the zebrafish *Hemogen* gene

The basic structure of the *Hemogen* gene of teleosts and mammals was also found to be highly conserved; four coding exons were separated by three introns (Fig. 1A), and two introns were found in the 5′-UTR. Two transcription start sites were predicted to occur within 2 kb upstream of the *Hemogen* start codon in zebrafish (Fig. 1A) and these appear to correspond to the hematopoietic- and testis-specific *Hemogen* promoters (noncoding exons 1H and 1T, respectively) described for mammals (Yang et al., 2003). Alignment of *Hemogen* genes from ten teleost species (Yates et al., 2016) revealed two conserved non-coding elements, CNE1 and CNE2, that overlapped with zebrafish exons 1T and 1H, respectively (Fig. 1A). We hypothesize that these elements function individually or together to regulate transcription of *Hemogen*.

### Transcription of the zebrafish *Hemogen* gene yields multiple mRNA isoforms

We confirmed transcription from both promoters in zebrafish by isolating and sequencing four splicing variants (Fig. 1C). Three isoforms were transcribed from the proximal promoter (exon 1H, Fig. 1A,C), each containing the same 5′-untranslated region (5′-UTR), compared to two corresponding mammalian transcripts. Alternative splicing of the second coding exon produced transcripts 1 and 2, which differ by four additional codons in the latter (Fig. 1C, red); the shorter version has not been described in mammals. Transcript 3 retained the entire third intron (156 bp), which introduced a premature translation-termination codon. A fourth isoform was transcribed from the distal promoter (1T) located ∼1.65 kb upstream of the translation start codon (Fig. 1A,C). Splicing of exons 1T and 1H to form the 5′-UTR of transcript 4 made use of canonical donor (AT-GT) and acceptor (AG-TT) splice sites.

### Teleost and mammalian Hemogen proteins differ markedly in size but share structural motifs

Teleost *Hemogen*-like genes encoded shorter proteins (194-289 amino acids) than the annotated *Hemogen* genes of mammals (417-827 amino acids), and the overall amino acid sequence similarity between teleost and mammalian orthologs was modest (18%-38%). Despite this heterogeneity in length and sequence, Hemogens of teleost fish and mammals shared predicted structural motifs, as shown in Fig. 1D,E for zebrafish (198 aa, 22 kDa) and human (484 aa, 55 kDa) orthologs, respectively. Their N-termini (zebrafish residues 1-74, human 1-78) were substantially conserved (51% sequence similarity; Fig. S1) and contained two predicted coiled-coil (CC) forming alpha-helices, the second of which was a putative nuclear localization signal (NLS) (Yang et al., 2001) (Fig. 1D; Fig. S1). By contrast, their C-termini (zebrafish residues 75-198, human 79-484) were weakly conserved in sequence (13% similarity), but both were rich in Pro and Glu residues (Figs S1 and S2), consistent with intrinsic disorder of these regions (Dyson and Wright, 2005). Furthermore, the C-termini shared modular structures – each was built of several 21-25 amino acid motifs, three in zebrafish and nine in humans, with distinct but related consensus sequences (PEXXXIAEXXXXXQEVXPQXXLVP and YSXEXYQEXAEPEDXSPETYQEIPX, respectively) (Fig. 1D,E; Figs S1 and S2). Thus, the size heterogeneity between zebrafish and human Hemogens was largely attributable to the number of repetitive segments contained within each.

Within the C-termini of teleost Hemogens, we identified a conserved acidic region (zebrafish residues 119-169, 35-49% similarity across ten species) that was similar to an acidic region of the mouse protein (Yang et al., 2001). Given the transactivation functions of Hemogen in humans (Zheng et al., 2014), we investigated whether the zebrafish and human proteins possessed TAD motifs based on the consensus sequences φφxxφ or φxxφφ, where φ is a bulky hydrophobic residue (Dyson and Wright, 2016). The acidic C-termini of both Hemogens contained one TAD motif. Four additional TAD motifs were distributed in other regions of the human protein (Fig. S1).

To assess the three-dimensional conformations of zebrafish and human Hemogens, although in a static context, we generated *ab initio* tertiary structural models with I-Tasser (Yang et al., 2015) using the best of ten predicted templates (Fig. 1E, see Materials and Methods). The structures for zebrafish and human Hemogens had template modeling scores (TM-scores) of 0.45 and 0.55, respectively, where a TM-score >0.3 indicates significantly different (*P*<0.001) from random structures (Xu and Zhang, 2010). When the two models were superimposed, amino acid sequences shared by human and zebrafish Hemogens showed 98% coincidence and a TM-score of 0.71. The N-termini of the zebrafish and human Hemogens presented exposed CC domains that may serve as binding sites for Gata1 (Zheng et al., 2014). The ‘disordered’ C-termini of Hemogens from zebrafish and humans were comprised of two distinct elements: proline-rich repeats (yellow) and an acidic, C-terminal repeat containing the TAD motif (brown) (Fig. 1E; Fig. S1). The former may coalesce as rigid linkers to extend the TAD motif to binding partners. These features are common to transcription factors, as epitomized by the structure of p53 (Wells et al., 2008).

### *Hemogen* expression tracks the ontogenetic progression of hematopoiesis in zebrafish

**Fig. 2.**
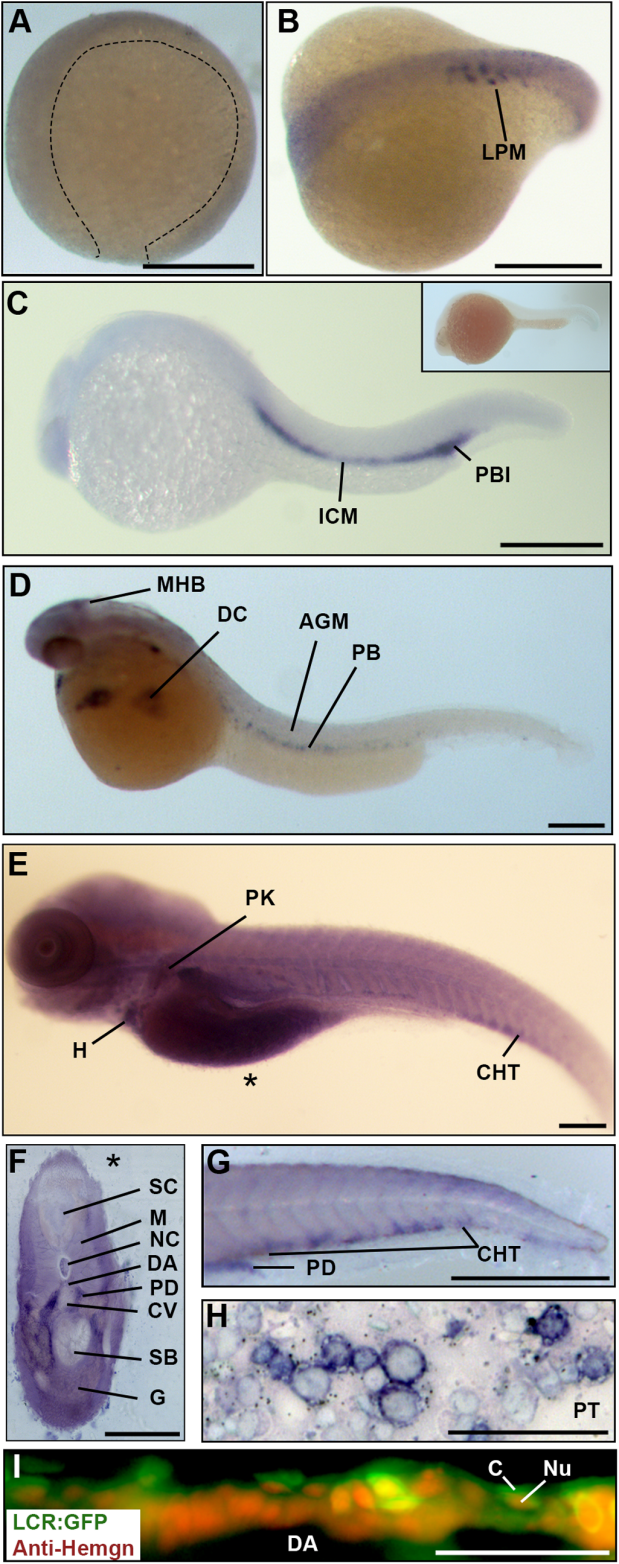
***Hemogen* expression in zebrafish embryos.** (A-H) Wild-type embryos, WISH. (A) Epiboly at 9 hpf. *Hemogen* expression was not detected. (B) Ten-somite stage. *Hemogen* transcripts along the lateral plate mesoderm (LPM). (C) 20 hpf. *Hemogen* staining in the intermediate cells mass (ICM) and posterior blood island (PBI). The inset shows a sense probe control. (D) 33 hpf. *Hemogen*-positive primitive erythrocytes of the peripheral blood (PB) exited the Ducts of Cuvier (DC) onto the yolk. Staining at the midbrain-hindbrain boundary (MHB) was observed. (E) 144 hpf. *Hemogen* expression in the caudal hematopoietic tissue (CHT) and pronephric kidney (PK) and in erythrocytes in the heart (H). The asterisk indicates the plane of the cross-section in panel F. (F) 144 hpf. Cross-section of embryo in panel E showing heavily stained pronephric ducts. (G) 48 hpf. Lateral aspect of tail. *Hemogen* transcripts in the CHT and pronephric tubule duct (PD). (H) Kidney touch print from adult fish. *Hemogen* expression was observed in proerythroblasts (ProE) and normoblasts (N) but not in erythrocytes (E). (I) 48 hpf. View of circulating EGFP+ erythrocytes in the dorsal aorta (DA) of *Tg(Lcr:EGFP)^cz3325Tg^* zebrafish after staining for Hemogen protein by indirect immunofluorescence. Hemogen (red signal) accumulated in nuclei (Nu) of erythrocytes whereas the cytoplasm (C) was marked by EGFP. Other abbreviations: AGM, aorta gonad mesonephros; PT, pronephric tubule; CV, caudal vein; DA, dorsal aorta; G, gut; M, myotomes; NC, notochord; SB, swim bladder; SC, spinal cord. Scale bars: (A-F) 250 µm; (G) 1 mm; (H) 100 µm; (I) 50 µm.

The spatial and temporal patterns of *Hemogen* expression were evaluated in zebrafish between 2 and 144 h post fertilization (hpf) by whole-mount *in situ* hybridization (WISH) (Fig. 2A-H). *Hemogen* transcripts were not apparent prior to somitogenesis (Fig. 2A) but first appeared at the ten-somite stage in punctate, intersomitic foci in the lateral plate mesoderm (LPM; Fig. 2B). By 20 hpf, *Hemogen* was expressed throughout the intermediate cell mass (ICM) and posterior blood island (PBI) (Fig. 2C), the sites of primitive hematopoiesis (Bertrand et al., 2007; Davidson and Zon, 2004). Primitive erythrocytes expressed *Hemogen* as they entered circulation at 33 hpf (Fig. 2D).

Definitive hematopoiesis in zebrafish embryos commences in the aorta gonad mesonephros (AGM) region at 30 hpf with the emergence of hematopoietic stem progenitor cells (HSPCs) that subsequently seed the caudal hematopoietic tissue (CHT) and the thymus (Murayama et al., 2006). By 144 hpf, HSPCs migrate from the CHT to establish a niche associated with the pronephric glomeruli (Bertrand et al., 2008). Although we did not detect *Hemogen* mRNA in the AGM (Fig. 2D), we observed strong expression in cells of the CHT at 48 and 144 hpf (Fig. 2E,G) and in the region of the pronephric glomeruli at 144 hpf (Fig. 2E,F). In the adult zebrafish kidney, *Hemogen* was strongly expressed in progenitor cells in the interstitial hematopoietic stem cell niche between pronephric tubules (Fig. 2H). *Hemogen* expression was robust in progenitors but absent in mature erythrocytes (Fig. 2H), whereas an anti-sense riboprobe for *βe1-globin* hybridized exclusively to mature erythrocytes but not to progenitor cells (data not shown).

Hemogen has been shown to function as a nuclear transcription factor in mammals (Zheng et al., 2014). To determine whether or not Hemogen is likely to play the same role in zebrafish, we examined *Tg(Lcr:EGFP)^cz3325Tg^* embryos at 48 hpf by indirect immunofluorescence microscopy using an antibody specific for Hemogen. *Tg(Lcr:EGFP)^cz3325Tg^* zebrafish have been used to track both primitive and definitive erythrocytes (Ganis et al., 2012). Fig. 2I shows that Hemogen accumulated in the nuclei (red signal) of GFP-labeled circulating erythrocytes in the dorsal aorta, thus, its role in transcription is likely to be conserved in zebrafish.

### Alternative promoters regulate Hemogen expression in zebrafish hematopoietic and reproductive tissues

**Fig. 3.**
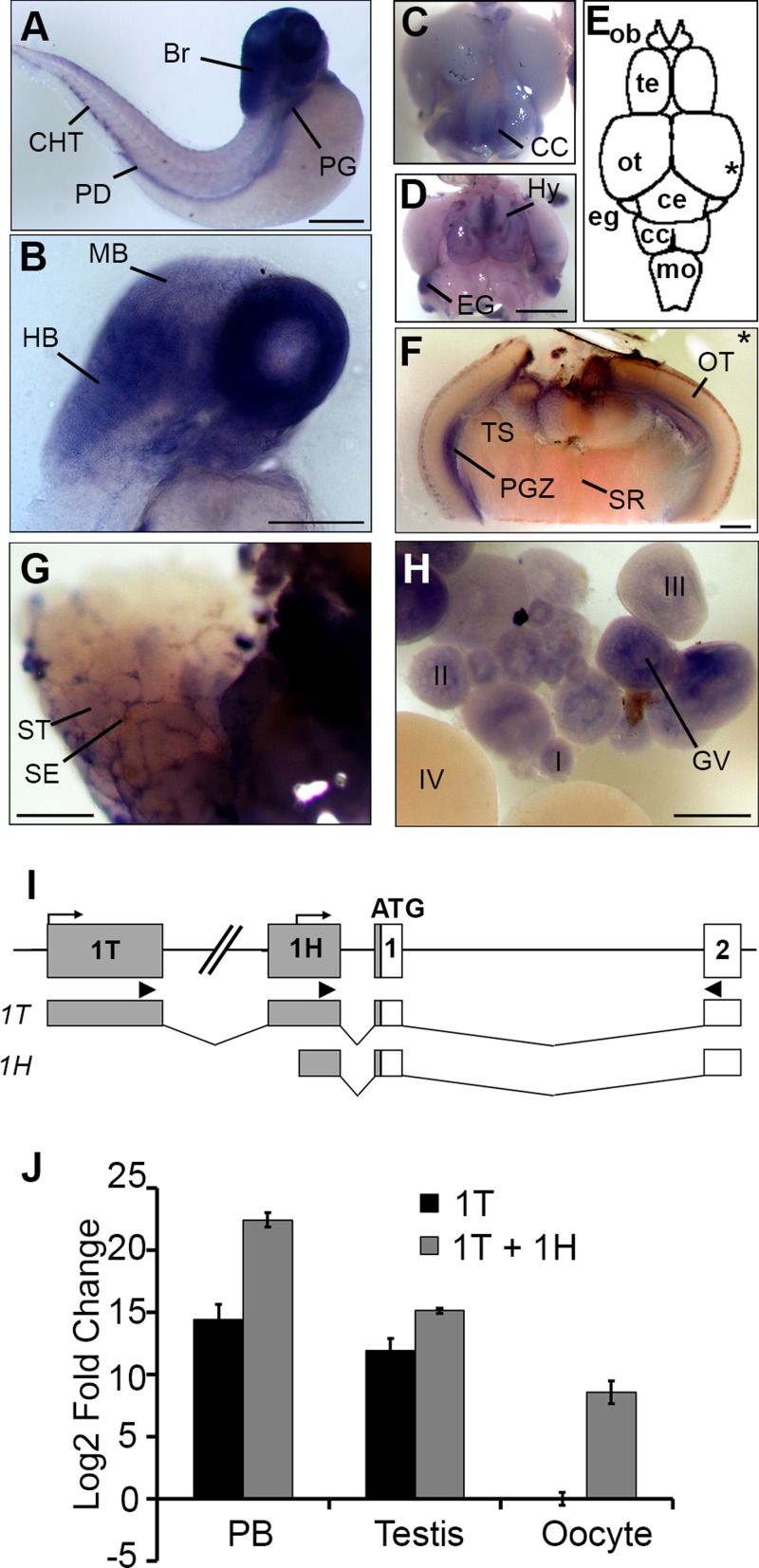
***Alternative* promoters drive *Hemogen*** **expression in hematopoietic and nonhematopoietic tissues in zebrafish.** WISH of wild-type embryos (A-B) and adult tissues (C-H). (A) 48 hpf. *Hemogen* expression in the pronephric kidney glomeruli (PG), pronephric tubule duct (PD), caudal hematopoietic tissue (CHT) and brain (Br). (B) 48 hpf. Section showing strong *Hemogen* expression in the hindbrain (HB) but at low levels in the midbrain (MB). (C,D) Dorsal (C) and ventral (D) views of the adult zebrafish brain after staining for *Hemogen* transcripts. CC, crista cerebellaris; Hy, hypothalamus; EG, eminentia granularis. (E) Schematic drawing of the dorsal view. *Hemogen* was highly expressed at the midbrain-hindbrain boundary within the EG, in the CC and in the Hy. The asterisk indicates the plane of the cross-section in panel F. (F) Section of the hindbrain showing *Hemogen* expression in the periventricular gray zone (PGZ). (G) *Hemogen* was expressed by Sertoli cells (SE) between the seminiferous tubules (ST) of the testes. (H) *Hemogen* was expressed in early (I-III) but not late (IV) stage oocytes. Transcripts accumulated around the germinal vesicle (GV). (I) Schematic of the *Hemogen* noncoding exons 1T and 1H (gray) upstream of the first coding exon (white); bent arrows, transcription initiation sites. Arrowheads mark primer binding sites for qPCR amplification of transcripts initiated from exons 1T or 1H. (J) Expression of transcripts from alternative promoters determined by qRT-PCR using RNA from blood, testes and ovaries of adult TU zebrafish. Expression in three biological replicates were normalized to *β-actin* and calculated relative to ovaries*.* Error bars represent the standard deviation. Transcription initiated from 1H must be inferred by difference [1H – 1T] because the 1H primers also amplified 1T transcripts. Other abbreviations: Ce, corpus cerebelli; MO, medulla oblongata; OB, olfactory bulb; OT, optic tectum; SR, superior raphe; Te, telencephalon; TS, torus semicircularis. Scale bars: 250 µm (A,B,F-H); 1 mm (C,D).

In zebrafish, we also detected *Hemogen* expression in the hindbrain and in the pronephric tubules of embryonic zebrafish between 30 and 48 hpf (Fig. 3A,B) and in adult zebrafish brain and reproductive tissues (Fig. 3C-H). The alternative *Hemogen* promoters found in zebrafish probably correspond to the hematopoietic and testis-specific *Hemogen* promoters of mammals (Yang et al., 2003). To quantify relative levels of transcription from each promoter in zebrafish (Fig. 3I), we performed qRT-PCR on total RNA from adult peripheral blood, testis and ovaries (Fig. 3J) using primer pairs specific for exons 1H and 1T. Because all of exon 1H was included in transcripts initiated from exon 1T, one must infer transcription from the proximal promoter by difference. Transcription from the proximal promoter was greatest in peripheral blood; the presence of transcripts from this promoter in testis and ovarian tissue may be due to contaminating blood RNA. The distal promoter was highly active in both peripheral blood and in testes but not in ovaries.

### Hemogen CNEs are predicted targets for transcription factors that regulate erythropoiesis and spermatogenesis

**Fig. 4.**
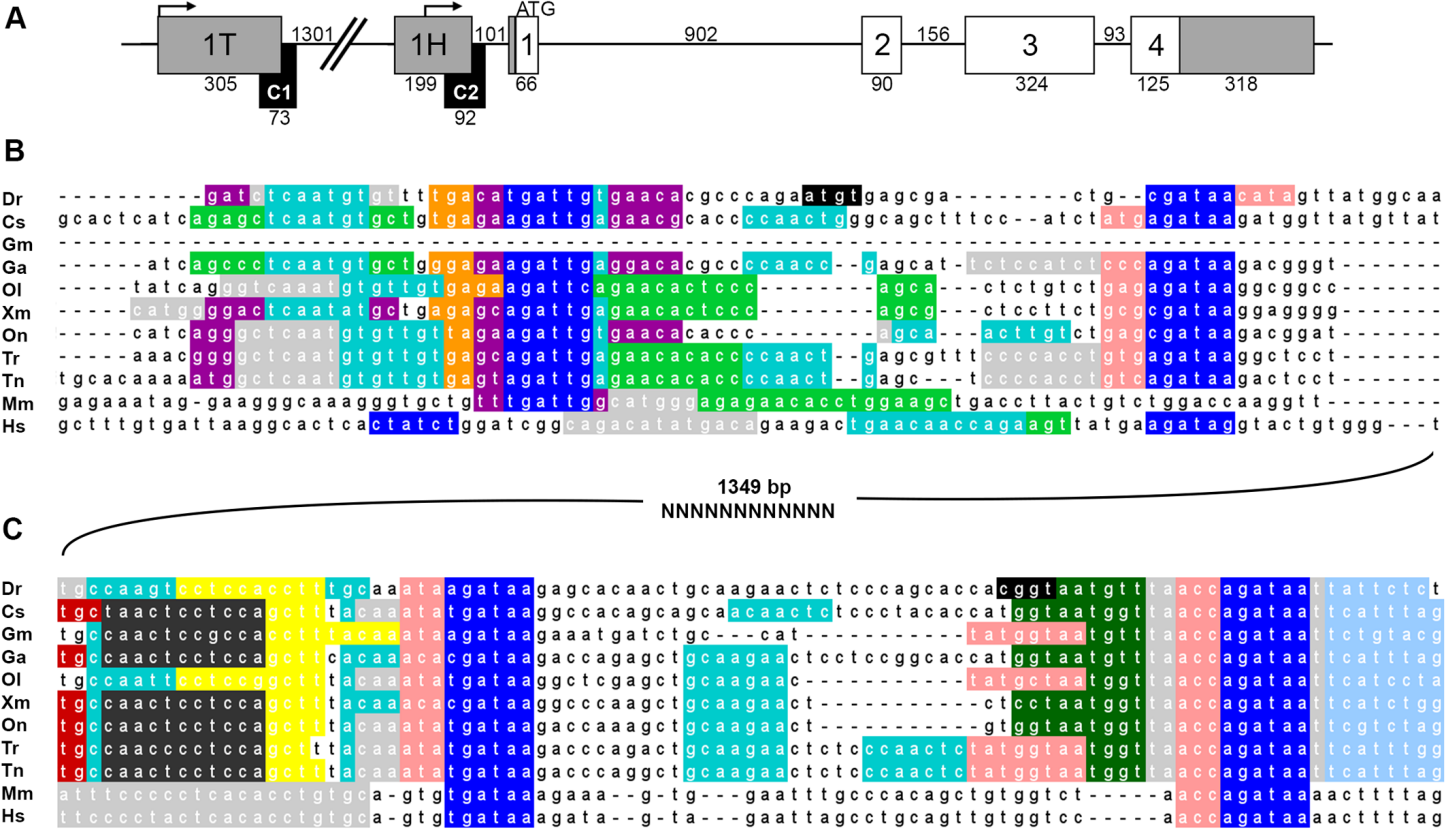
**Conserved elements in the zebrafish *Hemogen* promoter are predicted targets for transcription factors.** (A) Schematic of the zebrafish *Hemogen* gene. CNEs, black; coding exons, white; noncoding exons, gray; transcription initiation sites, bent arrows. Numbers indicate length in bp. (B,C) Sequence alignments of CNE1 and CNE2, respectively, from nine teleost species, mice and humans. ConTra software (Broos et al., 2011) predicted transcription factor binding sites for the Androgen receptor (light green), Brca1 (cyan), Foxl2 (pink), Gata1 (dark blue), Gfi1 (orange), HoxB4 (sky blue), Hnf1a (dark green), Klf4 (yellow), Myb (dark gray), P300 (red), Sox9 (purple) and Scl/Lmo2/Ldb1 complex (light gray). Splice donor sites are highlighted black. Species abbreviations: Dr, *Danio rerio*; Cs, *Cynoglossus semilaevis*; Gm, *Gadus morhua*; Ga, *Gasterosteus aculeatus*; Ol, *Oryzias latipes*; Xm, *Xiphophorus maculatus*; On, *Oreochromis niloticus*; Tr, *Takifugu rubripes*; Tn, *Tetraodon nigroviridis*; Mm, *Mus musculus*; Hs, *Homo sapiens*.

In teleosts, we identified two evolutionarily conserved non-coding elements, CNE1 and CNE2, that were tightly associated with exons 1T and 1H, respectively (Figs 1A and 4A). These elements may function as core promoters and/or enhancers to regulate transcription of the different *Hemogen* isoforms in zebrafish. To identify potential regulators of *Hemogen* transcription, we used ConTra v2 (Broos et al., 2011) to predict transcription factor binding motifs in the aligned *Hemogen* CNEs from two mammals and nine teleosts (Yates et al., 2016) (Fig. 4B,C). Each CNE contained binding motifs for transcription factors involved in erythropoiesis and/or spermatogenesis.

In zebrafish CNE2, two Gata1 binding sites, located +59 and +127 bp downstream relative to the transcription start site, aligned with Gata1 sites known to be active in the mammalian *Hemogen* promoter (Fig. 4C) (Yang et al., 2006). Each Gata motif was paired with a predicted E-box; this motif in *Hemogen* CNE2 is a known target of the Ldb1-erythroid-complex recruited by Scl (Soler et al., 2010). CNE2 also contained binding sites for Klf4, a driver of zebrafish primitive erythropoiesis (Gardiner et al., 2007), for Myb, a regulator of zebrafish definitive hematopoiesis (Soza-Ried et al., 2010), and for HoxB4, a regulator of *Hemogen* expression in mammalian hematopoietic stem cells (Jiang et al., 2010).

The distal CNE1 of teleosts possessed a similar suite of transcription factor binding motifs in roughly the same arrangement as the proximal CNE but with the notable addition of binding sites for Sox9 and the Androgen receptor (Fig. 4B), both of which play roles in zebrafish spermatogenesis (Hossain et al., 2008; Rodríguez-Marí et al., 2005). CNE1, like CNE2, contained pairs of E-box and Gata motifs downstream of the zebrafish transcription start site (+15 and +48 bp, respectively). CNE1 may function as an enhancer for the *Hemogen* gene and/or act as the core promoter for exon 1T.

### Hematopoietic and neural expression of Hemogen in zebrafish is dependent on Gata1 binding to the promoter CNEs

**Fig. 5.**
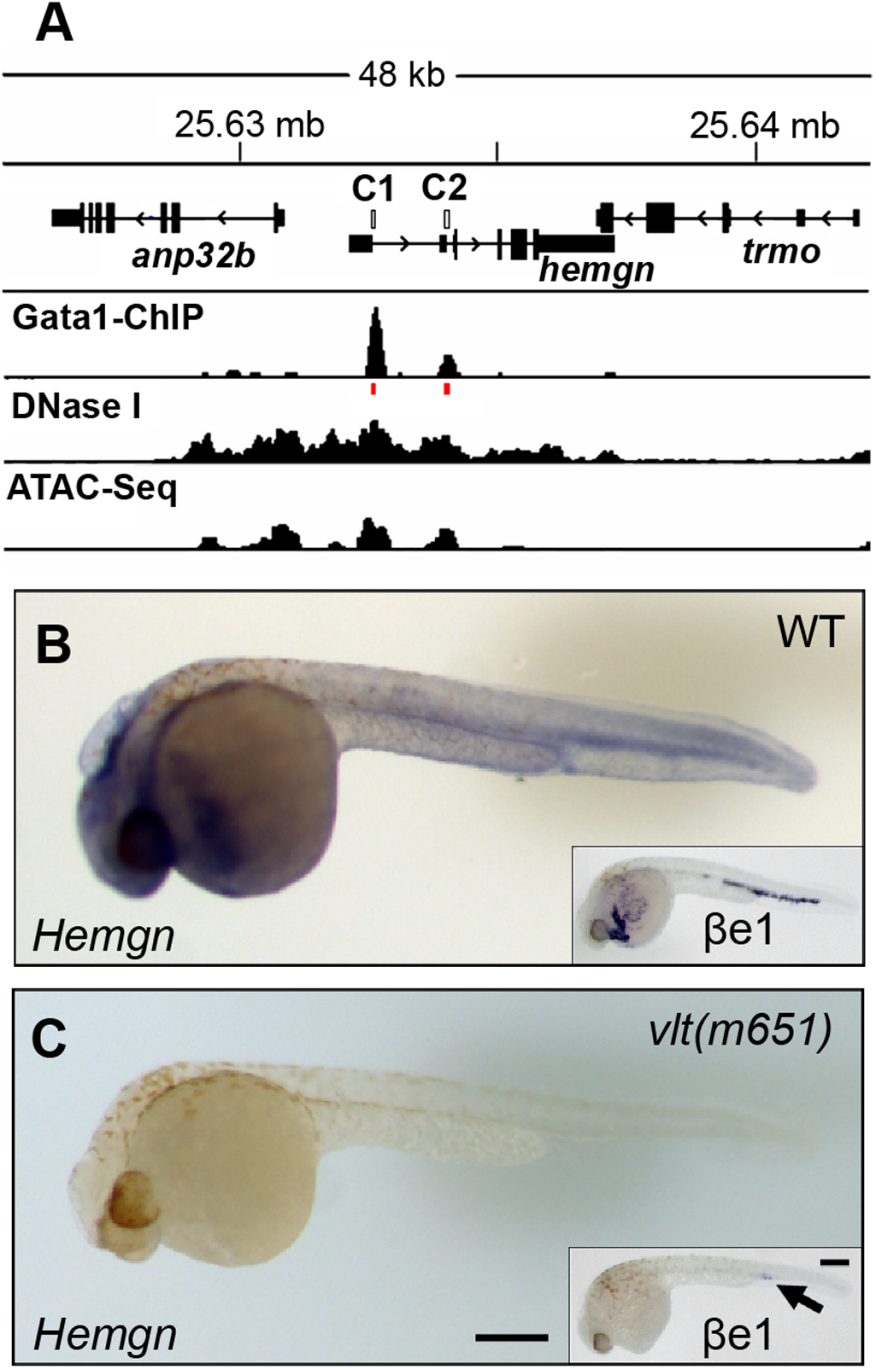
**Gata1 binds distal and proximal promoter elements to regulate *Hemogen* expression in zebrafish.** (A) Gata1 ChIP-sequencing showing enriched binding of Gata1 at CNE1 and CNE2 (C1 and C2, red lines) in the *Hemogen* promoter in adult zebrafish red blood cells (Yang et al., 2016). DNase-sequencing and ATAC-sequencing showing co-localization of the active chromatin regions (Yang et al., 2016). (B) *Hemogen* expression by WISH of wild-type (*n*=16/21) and (C) homozygous mutant (*n*=5/21) siblings (33 hpf) from in-crossed *Gata1*^+/−^ *vlt^m651^* mutants. Insets show *βe1-globin* expression in mutant (*n*=4/10) and wild-type (*n*=6/10) siblings. Scale bar: 250 µm (C).

In mammals, transcription of *Hemogen* from the proximal promoter is tightly regulated by Gata1 in hematopoietic cells (Yang et al., 2006). To investigate whether Gata1 regulates *Hemogen* in zebrafish, we analyzed a Gata1 ChIP-seq dataset that was generated to assess Gata1 activity in adult zebrafish erythrocytes (Yang et al., 2016). Fig. 5A shows that Gata1 bound to CNE1 and CNE2 at sites overlapping their Gata motifs (red lines), which indicates strongly that Gata1 is required for transcription of *Hemogen* in zebrafish. Corroboration that CNE1 and CNE2 were active chromatin regions was provided by ATAC-seq and DNase I hypersensitive site analysis (Yang et al., 2016) (Fig. 5A). Our data reveal that Gata motifs in CNE1, like those in CNE2, are important regulators of *Hemogen* expression in zebrafish erythrocytes.

We performed WISH to compare the expression of *Hemogen* and *Embryonic beta-globin* (*βe1-globin*) in embryos produced by the Gata1-null mutant, *vlad tepes* (*vlt^m651^*) (Lyons et al., 2002). At 33 hpf, *Hemogen* was expressed normally in circulating blood cells and in the hindbrain of wild-type siblings (Fig. 5B), and *βe1-globin* was abundant in the blood (Fig. 5B, inset). Homozygous *vlt^m651^* mutant siblings, by contrast, failed to express *Hemogen* in the blood and brain (Fig. 5C). This result mimicked the loss of *βe1-globin* in *vlt^m651^* mutants, with the exception that *βe1-globin* expression persisted in the PBI (Fig. 5C, inset), as has been demonstrated for *α1-globin, Scl* and *Gata1* (Jin et al., 2009).

### Tg(Hemgn:mCherry) zebrafish reveal the functions of the two *Hemogen* promoters

**Fig. 6.**
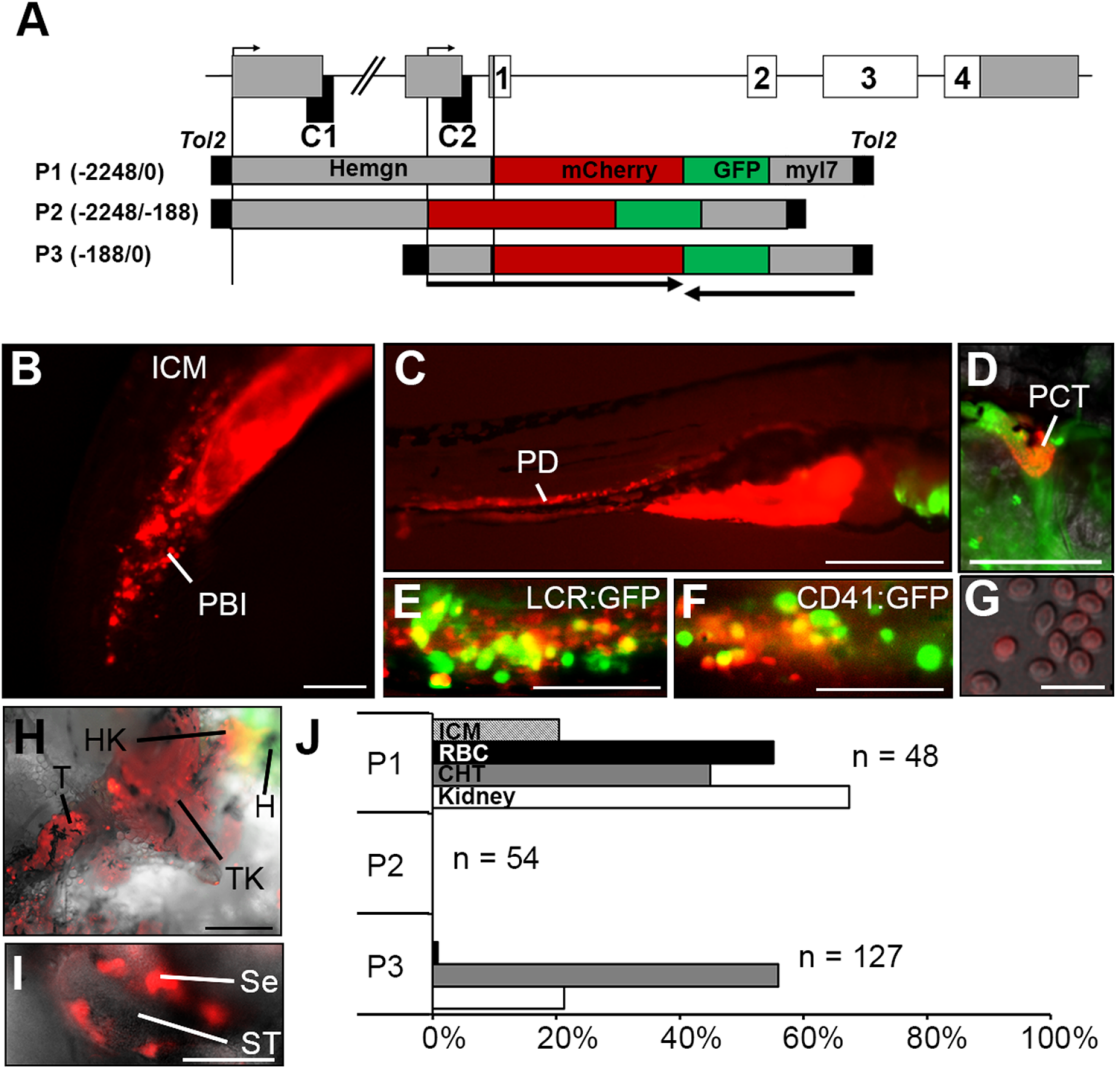
**Promoter elements have distinct roles in driving hematopoietic, renal and testicular expression of *Hemogen* in transgenic *Tg(Hemgn:mCherry)* zebrafish.** (A) Schematic of the zebrafish *Hemogen* gene. CNEs, black; coding exons, white; transcription initiation sites, bent arrows. Three *Tg(Hemgn:mCherry,myl7:EGFP)* transgenes driven by portions of the *Hemogen* promoter were transfected into one-cell TU embryos by *Tol2* transposase-mediated insertion. Numbers indicate length of promoter elements and arrows show gene direction. (B) 20 hpf. P1 transgene expression in the peripheral blood island (PBI). (C) 72 hpf. P1 transgene expression in the pronephric ducts (PD). (D) 5 dpf. P1 transgene expression in the proximal convoluted tubule (PCT). (E,F) 72 hpf. Co-localization of mCherry and EGFP in progenitors in the CHT of *Tg(Hemgn-P1:mCherry,Lcr:GFP)* or *Tg(Hemgn-P1:mCherry,CD41:EGFP)* zebrafish. (G) Transgene expression in mature erythrocytes from adult zebrafish. (H) Transgene expression in adult head kidney (HK), trunk kidney (TK) and tail kidney (T) near the EGFP+ heart (H). (I) Transgene expression in adult Sertoli cells (Se) that surround the seminiferous tubules (ST). (J) Proportion of embryos expressing transgenes P1, P2 or P3 in ICM, kidney, CHT and circulating primitive erythrocytes (RBC). Scale bars: 100 µm (B,D-F,I); 500 µm (C,H); 25 µm (G).

To determine the tissue-specific regulatory profiles of the two *Hemogen* promoters, we generated transgenic zebrafish embryos [*Tg(Hemgn:mCherry,myl7:EGFP*)] in which the *mCherry* reporter was controlled by the putative promoter elements (Fig. 6). The dual promoter, P1 (2248 bp), spanned the upstream, non-coding region to the *Hemogen* start codon and contained both CNEs. Transgenic fish were outcrossed to wild-type TU zebrafish and offspring with the strongest mCherry expression were selected as founders. In the early embryo, the P1 transgene drove expression of mCherry in primitive blood cells of the ICM and the PBI (20 hpf, Fig. 6B) and in primitive erythrocytes in circulation (Movie 1). Between 2 and 8 dpf, mCherry was expressed strongly throughout the pronephric ducts (Fig. 6C) and was present in the proximal convoluted tubule at 72 hpf (Fig. 6D). In adult transgenic fish, the head and trunk kidneys were positive for the reporter (Fig. 6H), as were Sertoli cells surrounding the seminiferous tubules of the testes (Fig. 6I). Therefore, the ∼2.2 kb P1 transgene contained all of the regulatory elements necessary to recapitulate *Hemogen* expression (Fig. 6B-I). We note that the dual promoter did not confer detectable ovarian or neural expression, which may require more distal sequences.

We found that the same expression profile was driven by the endogenous *Hemogen* promoter in embryonic zebrafish by using CRISPR/Cas9 technology to insert the *mCherry* gene (containing a polyadenylation motif) two codons downstream of, and in frame with, the *Hemogen* start codon (See Materials and Methods; Fig. S3A,C). Homology-directed integration of the transgene, confirmed by sequencing of the locus, produced mCherry+ cells in the CHT and in the kidney in 10% (*n*=15/150) of embryos at 3 dpf (Fig. S3B) and at a lower frequency in circulating RBC (*n*=3/150, data not shown).

To characterize hematopoietic cell lineages that express *Hemogen*, the P1 reporter plasmid was injected into embryos of *Tg(CD41:EGFP)^Ia2Tg^* or *Tg(Lcr:EGFP)^cz3325Tg^* zebrafish, which have been used to track hematopoietic progenitors (Lin et al., 2005) and primitive and definitive erythrocytes (Ganis et al., 2012), respectively. We did not observe *mCherry* expression in the AGM, in the thymus, or in CD41+ HSPCs colonizing the thymus or pronephros (Bertrand et al., 2008). However, the reporter was strongly expressed in a subset of LCR+ erythroid and CD41+ myeloid-biased progenitors in the CHT (Fig. 6E,F), a tissue that supports myelopoiesis (Gekas and Graf, 2013; Medvinsky et al., 2011). This lends support to previous findings that *Hemogen* is a marker and promoter of myeloerythroid, but not lymphoid, lineages (Li et al., 2007; Lu et al., 2001). Maturing mCherry+ primitive progenitors peaked in brightness just prior to leaving the caudal plexus and entering circulation at 72 hpf (observed by time-lapse imaging; data not shown). However, mature definitive erythrocytes expressed little mCherry in adult transgenics (Fig. 6G), which supports prior observations that *Hemogen* expression is limited to primitive erythrocytes and immature definitive progenitors (Lu et al., 2001).

### Hemogen promoters have different functions in primitive and definitive erythropoiesis in zebrafish

We evaluated the separate and combined contributions of the two *Hemogen* promoters, including CNE1 or CNE2, to the observed tissue-expression profiles by injecting wild-type embryos with one of three *Tg(Hemgn:mCherry,myl7:EGFP)* reporter constructs in which mCherry expression was driven: (1) by the dual promoter (P1); (2) by a 2 kb fragment (P2) containing the distal promoter including CNE1; or (3) by a 188 bp fragment (P3) containing the proximal promoter including CNE2 (Fig. 6A). Transgenic embryos were screened for EGFP+ hearts, and *mCherry* transcription was confirmed by RT-PCR and sequencing.

mCherry fluorescence was examined in four cell types: (1) erythroid progenitors in the ICM at 1 dpf, (2) primitive erythrocytes in the peripheral blood at 3 dpf, (3) erythroid progenitors in the CHT at 3 dpf and (4) renal cells of the kidney tubules at 3 dpf. Fig. 6J shows that the dual promoter (P1) supported strong expression of the mCherry reporter in erythroid cells of the ICM and peripheral blood (RBC), in the CHT and in renal cells of the kidney. By contrast, the distal promoter (P2 construct) containing CNE1 failed to drive reporter expression in these tissues. Finally, the proximal promoter (P3 construct) containing CNE2 alone produced strong expression of the reporter in the CHT and in kidney cells but was not active in cells of the ICM and peripheral blood. Together, these results indicate that the proximal promoter containing CNE2 is necessary and sufficient to drive expression in definitive hematopoiesis and in the kidney, whereas the full 2.2 kb sequence including both promoters and CNEs is required in primitive erythropoiesis.

### Morpholino knockdown of Hemogen protein expression partially disrupts erythropoiesis in zebrafish

**Fig. 7.**
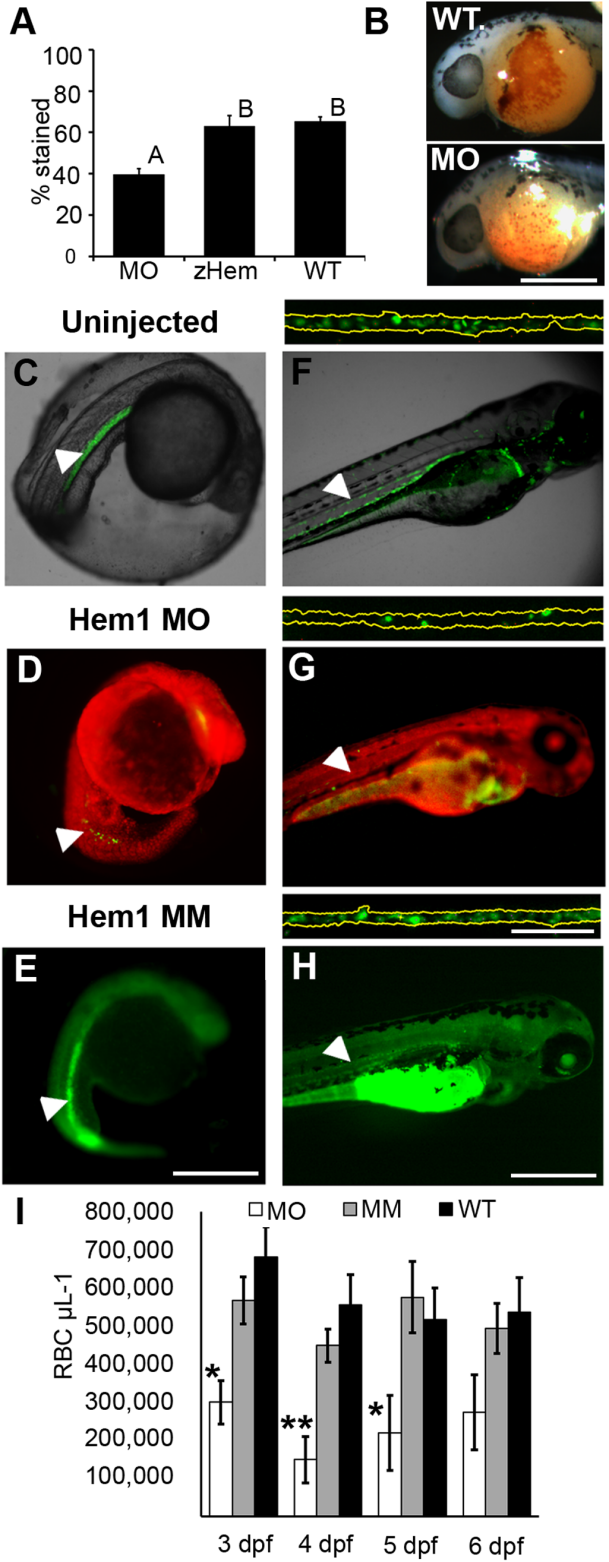
**Morpholino targeting of *Hemogen* inhibits erythropoiesis in embryonic zebrafish.** Embryos were injected with 2 to 4 ng antisense MO targeted to the first 25 coding nucleotides of *Hemogen*. (A-B) *O*-dianisidine staining of erythrocytes was decreased in morphants (MO) relative to wild-type embryos (WT) or embryos rescued with 500 pg synthetic *Hemgn* mRNA (zHem) at 24 hpf. (ANOVA, Tukey post hoc test, *P*<0.001). (C-E) Live wild-type (C), Hem1 MO-injected (D) and Hem1 mm mismatch MO-injected (E) *Tg(Lcr:EGFP)^cz3325Tg^* embryos at 20 hpf. Morphants showed decreased EGFP expression in the ICM compared to the wild-type and mismatch MO controls. (F-H) Live wild-type (F), Hem1 MO-injected (G) and Hem1 mm MO-injected (H) embryos at 72 hpf. Morphant embryos have fewer EGFP+ cells in circulation compared to the two controls. The dorsal aortas of embryos (insets above F-H) were magnified 20× to permit quantitation of EGFP+ erythrocytes. Background red (D,G) and green (E,H) fluorescence was generated by the fluorescent labels on the MOs. (I) *In vivo* flow quantitation of EGFP+ erythrocyte concentrations between 3 and 6 dpf in Hem1-injected (*n*=9,7,7,7), Hem1mm-injected (*n*=13,14,11,11) and uninjected (*n*=5,10,10,9) embryos. Data shown as means±s.e.m. (*P≤0.05, **P≤0.001, ANOVA, Tukey-Kramer post hoc test). Arrowheads show notochord kinking. Scale bars: 500 µm (A-F); 100 µm (inset).

To perturb *Hemogen* function in zebrafish, we first injected wild-type zebrafish embryos at the one-cell stage with an antisense morpholino oligonucleotide (MO), Hem-1, targeted to the translation start codon of the *Hemogen* transcript (Hem1). MO treatment significantly reduced Hemogen protein levels by 19% at 33 hpf (Student's *t*-test, *P*<0.05, Fig. S4A,B) and steady-state levels of *βe1-globin* mRNA at 3 dpf (Student's *t*-test, *P*<0.05, Fig. S4C). At 24 hpf, 61% of morphants were anemic compared to 35% of uninjected zebrafish (Fig. 7A,B). Red cell levels were restored to wild-type by co-injection of the MO with 500 pg of synthetic zebrafish *Hemogen* mRNA containing silent mutations in the MO target site. Both the uninjected and rescue treatments differed significantly from the MO treatment (ANOVA, Tukey post hoc test, *P*<0.001, Fig. 7A).

We used *Tg(Lcr:EGFP)^cz3325Tg^* zebrafish to visualize the red blood cell population in Hem1-treated morphants from 0 to 6 dpf. Control embryos were injected with a 5 bp mismatch MO (Hem1mm) or were uninjected. At 20 hpf, EGFP+ erythrocytes appeared to be reduced in the ICM/PBI of 75% of Hem1 morphants (*n*=56) but not in mismatch or uninjected control embryos (*n*=14 and 63, respectively) (Fig. 7C-E). At 2 dpf, morphant embryos had few erythrocytes in circulation compared to controls (Fig. 7F-H, Movie 2). Using quantitative *in vivo* flow analysis (Fig. 7I), we found that morphant embryos at 3-5 dpf had fewer than 50% of the circulating EGFP+ erythrocytes as the uninjected and Hem1mm-injected controls, whereas the controls did not differ statistically from each other (ANOVA, Tukey-Kramer post hoc test, *P*<0.05).

### A conserved C-terminal domain in Hemogen is required for hematopoiesis and prevents apoptosis in embryonic tissues

**Fig. 8.**
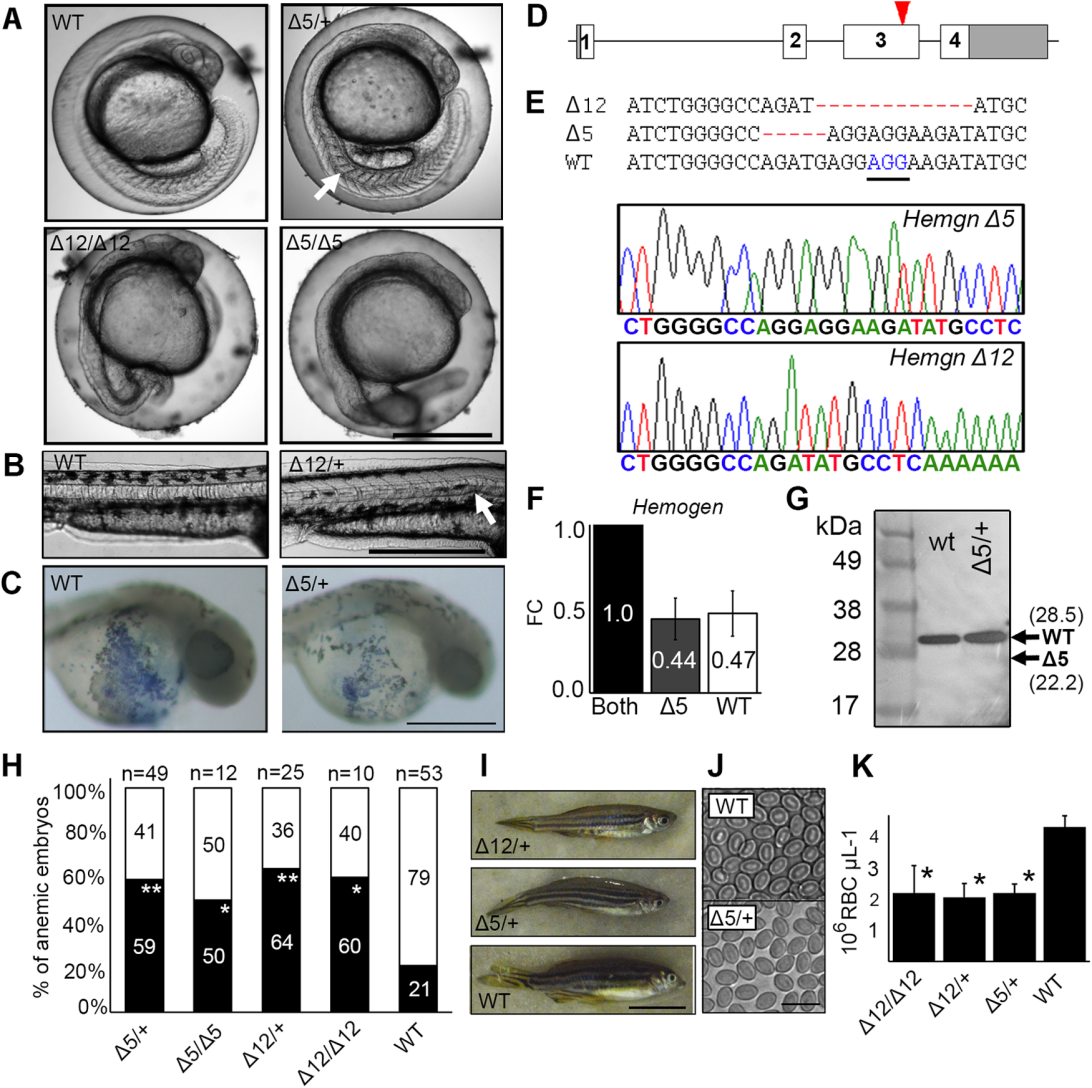
**CRISPR/Cas9 mutagenesis of the third exon of zebrafish *Hemogen* reduces primitive and definitive erythropoiesis.** Embryos were injected with Cas9 mRNA and a guide RNA to establish lines with mutations in exon three of zebrafish *Hemogen*. (A) 20 hpf. Representative wild-type and mutant siblings with notochord defects (arrow). (B) 48 hpf. Mutant *Δ12* embryos with an in-frame deletion showing kinked notochords (arrow). (C) 24 hpf. Wild-type and *Δ5/+* mutant embryos stained with diaminofluorene. Production of erythrocytes was reduced in heterozygotes. (D) Schematic of CRISPR/Cas9 target in the third exon (red arrowhead) of zebrafish *Hemogen*. (E) Sequences of founder mutations aligned at the CRISPR target site: *Δ5* (*Hemgn^nuz2^*); *Δ12* (*Hemgn^nuz4^*). The sequence traces show the *Δ5 and Δ12* mutant alleles. PAM, blue and underlined; Δ, deletions (highlighted in red). (F) Relative expression of wild-type and *Δ5* transcripts in blood from single adult, heterozygous *Hemgn^nuz2/+^* mutants determined by qRT-PCR with allele specific primers. Three biological replicates were normalized to β-actin. Error bars represent the standard deviation. (G) Western blot of Hemogen in pooled 33 hpf wild-type embryos or pooled embryos from a *Δ5 Hemgn^nuz2/+^* heterozygous in-cross. We calculated that the protein would run 6.5 kDa above its molecular weight at 28.5 kDa because of its high acidic composition (Guan et al., 2015). Arrows show the calculated sizes of wild-type and truncated alleles. (H) Proportion of genotyped mutants and wild-type sibling embryos at 2 dpf that were anemic (black) or phenotypically normal (white) (*P≤0.05, **P≤0.005, Chi square). (I) Wild-type and mutant zebrafish heterozygous for the *Δ5* and *Δ12* alleles. (J) Red blood cells from adult *Hemgn^nuz2/+^* mutant zebrafish and wild-type siblings. (K) Erythrocyte counts in adult heterozygous *Hemgn^nuz2^* (*Δ5, n*=12), heterozygous *Hemgn^nuz4^* (*Δ12*, *n*=4) mutants, homozygous *Hemgn^nuz4^* (*Δ12*, *n*=2) mutants and wild-type (*n*=9) siblings (*P≤0.05, ANOVA, Tukey post hoc test). Scale bars: 500 µm (A-C); 50 mm (I); 20 µm (J).

The function of the putative C-terminal transactivation domain of zebrafish Hemogen was investigated using CRISPR/Cas9 mutagenesis. We generated zebrafish lines with mutations in the conserved region near the end of the third coding exon of *Hemogen,* immediately downstream of the TAD motif (Fig. 8A-D; Fig. S1). Founders (F0) were out-crossed to wild-type TU zebrafish and mutant alleles were genotyped in the F1 generation by high resolution melting analysis and by sequencing the locus (Fig. 8E; Fig. S1). One line, *Hemgn^nuz2^*, had a 5 bp deletion (*Δ5*) that produced a frameshift mutation, thereby introducing a premature stop codon (Fig. 8E; Fig. S1). PolyA-tailed transcripts of the *Δ5* allele were detected at equivalent steady-state levels relative to the wild-type allele in peripheral blood from individual adult heterozygotes (Fig. 8F). Western blot analysis revealed, however, that truncated Hemogen protein was almost undetectable in peripheral blood from single heterozygous adults (data not shown) and in pooled 33 hpf embryos from a heterozygous in-cross (Fig. 8G). Therefore, if the truncated *Hemgn^nuz2^* transcripts were translated, then the protein must have been rapidly degraded. The second line, *Hemgn^nuz4^*, contained an in-frame 12 bp deletion (*Δ12*), which deleted an acidic cluster (EEED) in the last repeat that is conserved in teleost species (Fig. S1). In contrast to *Δ5* mutants*,* Hemogen protein was detected in the blood of homozygous Δ12 adults by western blot (data not shown).

To evaluate the effects of the mutant *Hemogen* alleles on erythropoiesis during development, we examined embryos from mutant crosses by microscopy and genotyped them between 20 and 48 hpf (Fig. 8A-C); mutant genotypes were recovered near the expected Mendelian ratios (Fig. S5A), but homozygous *Δ5 hemgn^nuz2^* mutants could not be raised to adulthood. To classify the mutants, we assessed the relative numbers of blood cells and relative concentrations of hemoglobin beginning at 2 dpf (Ransom et al., 1996). Embryos from a heterozygous in-cross were scored for hypochromic blood (paler blood) and decreased numbers of circulating cells on the yolk sac and in the vasculature. Erythrocyte levels were reduced to about 25-75% of normal levels in frameshift *Hemgn^nuz2/+^* mutants (*n*=8) at 24 hpf compared to wild-type siblings (*n*=7) (Fig. 8C). At 48 hpf, 59% of heterozygous (*n*=49) and 50% of homozygous (*n*=12) *Hemgn^nuz2^* mutants had reduced numbers of circulating erythrocytes (Fig. 8H, Movie 3) and homozygotes could be distinguished by their more severe anemia. Comparable numbers of anemic individuals were observed for heterozygotes and homozygotes of the *Δ12 Hemgn^nuz4^* allele: 64% (*n*=25) and 60% (*n*=10), respectively (Fig. 8H). In all cases, the proportion of anemic mutant embryos was significantly different from that for wild-type (*P≤0.05, **P≤0.005, Chi square).

Erythrocyte levels in adult mutants were partially suppressed in heterozygotes. *Hemgn^nuz2/+^* and *Hemgn^nuz4/+^* adults gave average erythrocyte counts of 2.2±1.0×10^6^ cells µl^−1^ and 2.1±0.8×10^6^ cells µl^−1^, respectively, whereas wild-type zebrafish had 4.3±1.0×10^6^ cells µl^−1^ (Fig. 8J,K). Homozygous *Δ12 Hemgn^nuz4^* gave average erythrocyte counts of 2.2±1.2×10^6^ cells µl^−1^ (Fig. 8K). Taken together, the erythroid defects of embryonic and adult zebrafish carrying the CRISPR-generated mutant alleles support the conclusion that the conserved C-terminus of Hemogen functions as a TAD, but the mechanism of action of these mutations remains to be determined.

Both the *Δ5* and *Δ12* mutant *Hemogen* alleles also caused mild to severe developmental defects in the nototchord and the trunk of heterozygotes and homozygotes (Fig. 8A,B; Fig. S5B). Embryos had kinked notochords and exhibited increased cellular refractility consistent with apoptotic cell death. Elevated apoptotic cell death was apparent in *Hemgn^nuz2/+^* mutants as detected by staining with Acridine Orange (Fig. S5C). Apoptosis occurred throughout the embryo, including sites of embryonic hematopoiesis. Nevertheless, viable heterozygotes for both alleles could be raised to adulthood; they were slightly smaller than wild-type siblings (Fig. 8I). Impaired growth was significant in homozygous *Δ12 Hemgn^nuz4^* adult mutants (Student's *t*-test, *P*=0.04, *n*=3; Fig. S5D,E).

## DISCUSSION

The zebrafish is a compelling model for understanding the pleiotropic functions of Hemogen in the context of vertebrate development. Our results show that zebrafish Hemogen is considerably smaller than its human ortholog, a distinction true for teleost and mammalian Hemogens in general. Hemogen is expressed in multiple zebrafish tissues from the early embryo to the adult under the control of at least two promoters. Both primitive and definitive erythropoiesis are affected by depletion of Hemogen and by targeted mutation of a putative, C-terminal TAD. The transgenic and mutant zebrafish lines that we have generated will contribute to a mechanistic understanding of this important transcription factor.

### Hemogen – small or large, it's built of related modules and has a conserved role in erythropoiesis

We show that the divergent Hemogens of zebrafish and human are largely, but not entirely, built of 21-25 residue repeats; the number of repeats largely determines protein size. The repeat consensus sequences are distinct, but they appear to have evolved from an 8-10 amino acid core motif (Fig. S2). Although all repeats are acidic (Fig. S2), the terminal repeat of each Hemogen is particularly so (>38% Asp and Glu for zebrafish, >29% for human), and these repeats contain TAD motifs. Together, these features suggest that Hemogens possess flexible, intrinsically disordered TADs, as is true of many transcription factors (e.g. p53, HIF-1α, NF-κB, etc.). The multivalent structure of Hemogen provides opportunities for cooperative binding to single or multiple protein partners, including P300 (Zheng et al., 2014).

Hemogen interacts with a variety of proteins to stimulate the transcription of genes involved in terminal erythroid differentiation and other processes. In humans, Hemogen contributes to transcription of erythroid genes in part by recruiting P300 to acetylate and activate Gata1 (Zheng et al., 2014). Our results show that nonsense (*Δ5*) and deletion (*Δ12*) alleles of *Hemogen* vicinal to the zebrafish TAD motif cause significant reductions of erythrocyte levels in embryos and adults. The *Δ12* allele may be hypomorphic, but we have not determined whether the protein that is expressed has reduced activity.

### Hemogen – targeted mutation of the acidic C-terminus impairs erythropoiesis, but not completely

Our CRISPR-generated zebrafish mutant lines show that nonsense (*Δ5*) and deletion (*Δ12*) alleles of *Hemogen* caused a decrease in erythrocyte levels in embryos and adults. However, these phenotypes were incompletely penetrant; in both heterozygous and homozygous *Hemogen* mutants the proportion of anemic embryos was 50-65%, compared to 20% for wild types. If *Hemogen* were essential for erythropoiesis, one would anticipate an erythroid-null phenotype for homozygous mutants, as observed for the *Gata1* mutant, *vlad tepes^m651^* (Lyons et al., 2002). Rather, the *Hemogen* phenotype resembles the variable reduction of red cells in zebrafish *zinfandel* (*zin^te207^*) mutants that harbor a mutation in a regulatory region at the globin locus (Ransom et al., 1996), a known target of both Hemogen and Gata1 transcription factors (Zheng et al., 2014). Loss of Hemogen in zebrafish contributes to decreased expression of *Embryonic beta-globin* (Fig. S4), which may explain the hypochromic state of *Hemogen* mutants.

The most plausible explanation for the incomplete penetrance of anemia in *Hemogen* mutants is the phenomenon of genetic compensation, which may occur when genes are knocked out as opposed to knocked down (El-Brolosy and Stainier, 2017; Rossi et al., 2015). Although the mechanisms are poorly understood, genetic compensation entails changes in gene expression (e.g. upregulation of paralogous genes or functionally related genes) that at least partially offset the phenotype caused by the mutant protein. Compensation through elevated expression of other erythroid co-activators is an attractive possibility that might maintain erythrocyte production in *Hemogen* mutants. The functional loss of Hemogen could be mitigated by Gata1 homodimerization and/or by direct recruitment of CBP/P300, both of which enhance Gata1 activity (Ferreira et al., 2005; Nishikawa et al., 2003).

### Similar design and regulation of *Hemogen* and *Gata1* genes

Comparison of the expression of *Hemogen* and of *Gata1* throughout zebrafish development reveals a remarkable degree of overlap in tissue and cellular specificity. For example, *Gata1* mRNA appears in cells of the LPM at the two-somite stage (Detrich et al., 1995), immediately prior to the onset of *Hemogen* expression at ten somites. Furthermore, *Hemogen* and *Gata1* are co-expressed in primitive erythrocytes and definitive hematopoietic progenitors (Ferreira et al., 2005; Lu et al., 2001), in Sertoli cells (Nakata et al., 2013; Wakabayashi et al., 2003) and at the midbrain-hindbrain boundary (Volkmann et al., 2008). Interestingly, both *Hemogen* and *Gata1* genes possess hematopoietic- and testis-specific promoters (Wakabayashi et al., 2003)*.* The temporal and spatial co-incidence of *Hemogen* and *Gata1* expression almost certainly results from their similar regulatory architectures and also through regulatory crosstalk. Our results and studies conducted by others (Ding et al., 2010; Yang et al., 2006; Zheng et al., 2014) indicate that reciprocal transcriptional activation of *Hemogen* and *Gata1* may form a positive feedback loop that drives erythropoiesis.

Strikingly, the two CNEs of *Hemogen* are organized like, and have the same functions as, the distal and proximal enhancers of the *Gata1* gene (McDevitt et al., 1997; Onodera et al., 1997; Suzuki et al., 2009). The proximal *Gata1* promoter functions exclusively in definitive erythropoiesis (McDevitt et al., 1997), as does CNE2 of zebrafish *Hemogen*. In contrast, transcription of *Gata1* in primitive erythrocytes requires both the proximal promoter and a distal enhancer comparable to *Hemogen* CNE1 (McDevitt et al., 1997). Fig. S6 presents a model for the transition from primitive to definitive hematopoiesis based on chromatin looping at the *Hemogen* locus. We propose that the transition from primitive to definitive erythropoiesis involves a switch from a loop conformation to a linear conformation, mediated by the Gata1/Ldb1-complex at erythroid transcription factories (Osborne et al., 2004; Schoenfelder et al., 2010). This model may also apply to the *Gata1* enhancer, which is another known target of the Ldb1-complex (Love et al., 2014). The zebrafish lines produced in this study may help clarify the cell-specific *Hemogen* expression profile driven by different Gata1-containing complexes and the functions of Hemogen in different cell types.

## MATERIALS AND METHODS

### Fish husbandry

Wild-type (SAT, AB, TU) zebrafish (*Danio rerio*), the transgenic lines *Tg(Lcr:EGFP)^cz3325Tg^* (Ganis et al., 2012) and *Tg(CD41:EGFP)^Ia2Tg^* (Traver et al., 2003) and the mutant *vlad tepes^m651^* (Lyons et al., 2002) were all generously provided by Dr Leonard I. Zon (Howard Hughes Medical Institute and Harvard Medical School, Boston). Animal procedures were carried out in full accordance with established standards set forth in the *Guide for the Care and Use of Laboratory Animals* (8th Edition). The animal care and use protocol for live zebrafish embryos was reviewed and approved by Northeastern University's Institutional Animal Care and Use Committee (Protocol No. 15-0207R). The animal care and use program at Northeastern University has been continuously accredited by AAALAC Int. since 22 July 1987 and maintains the Public Health Service Policy Assurance Number A3155-01.

### Cloning and sequence analysis of zebrafish *Hemogen* cDNAs

Total RNA was isolated from wild-type AB zebrafish embryos and adult tissues (kidney, blood, brain, ovary, intestine) using TRI reagent (Sigma-Aldrich; T9424) and the Ribopure Kit (Ambion, Foster City, USA; AM1924). Total cDNA was produced from mRNA using M-MuLV reverse transcriptase [New England Biolabs (NEB), Ipswich, USA; M0253S] and an oligo(dT)_23_ primer. *Hemogen* cDNA was amplified by PCR from total cDNA with 1 µM primers (Table S1). The amplification program was 35 cycles of 98°C for 10 s, 57°C for 10 s and 72°C for 30 s. PCR products were cloned into the pGEM-T Easy vector (Promega, Madison, USA; A1360), plasmids were transformed into 5-α competent cells (NEB; C2987H), recombinant plasmids were id entified by blue/white screening and purified with the Wizard Plus SV Miniprep Kit (Promega; A1330) and inserts were sequenced by GENEWIZ, Inc. (Cambridge, USA).

### Bioinformatic comparison of vertebrate *Hemogen* genes and Hemogen proteins

We utilized the murine gene nomenclature for comparing orthologs from different vertebrate species. We used Blast+ (Altschul et al., 1990) to identify *Hemogen* in the zebrafish genome (assembly GRCz11) (Howe et al., 2013). Chromosomal synteny comparisons were performed using the Synteny Database with a sliding window of 200 genes (Catchen et al., 2009) and Ensembl Genomes v74 (Kersey et al., 2016). *Hemogen* promoter alignments were obtained from whole genome alignments for ten teleost species (ENSEMBL v74) (Yates et al., 2016). Transcription factor binding motifs were predicted using the program ConTra with the default similarity matrix of 0.75 (Broos et al., 2011). Transcription start sites were predicted using NNPP v2.2 with a score cutoff of 0.98 (Reese, 2001).

Protein domains in zebrafish were identified using annotated human Hemogen (Yang et al., 2001), or they were predicted using HHpred (Soding et al., 2005) and the Conserved Domain Database (CDD) (Marchler-Bauer et al., 2015). Peptide repeats were predicted with RADAR (Heger and Holm, 2000). The 9aaTAD Prediction Tool was first used to predict transactivation domain (TAD) motifs, starting with low stringency DFx repeats (Piskacek et al., 2016). These were then culled by φφxxφ or φxxφφ criteria, where φ is a bulky hydrophobic motif (Dyson and Wright, 2016). We refer to the latter five amino acid consensus sequences as ‘TAD motifs’, in contrast to larger, functionally defined ‘transactivation domains’ (TADs). *Ab initio* tertiary structure models were created for zebrafish and human Hemogen proteins with I-Tasser (Yang et al., 2015) based on the X-ray structure for the secretory component of Immunoglobulin A (PDB:3chnS), which was the best of ten predicted structural templates determined by LOMETS (Wu and Zhang, 2007). The 3D models were superimposed using TM-align (Zhang and Skolnick, 2005) and Geneious version R10 (Kearse et al., 2012).

### MO knockdown of *Hemogen* in zebrafish and rescue of the morphant phenotype

The antisense MO Hem1 (5′-TCTCTTTCTCCAACGGGTCTTCCAT-3′), which targets the first 25 base pairs of the zebrafish *Hemogen* open reading frame, was designed according to the manufacturer's instructions (Gene Tools LLC, Philomath, USA). The control MO (Hem1mm; 5′-TCTgTTTgTCCAtCGGcTCTTCgAT-3′) targeted the same sequence but contained five mismatched bases to prevent efficient binding to *Hemogen* mRNA. MOs were labeled with lissamine or fluorescein so that the quality of injections could be monitored by fluorescence microscopy. MOs were injected (2-8 ng) into embryos at the single-cell stage using a PLI-100 Picoinjector (Medical Systems Corporation, Greenvale, USA; 65-0001) and a micromanipulator (Narishige, Amityville, USA; MN-151). Injected embryos were sampled from 0 to 6 dpf for subsequent analyses.

Rescue of the morphant phenotype was tested by co-injection of the Hem1 MO with 500 pg synthetic zebrafish *Hemogen* mRNA transcribed from a zebrafish *Hemogen* cDNA cloned into pGem-T Easy (Promega). Primers (Table S1) introduced five silent mutations within the MO target site. The clone was digested with *Spe1* and mRNA was transcribed, capped and polyadenylated *in vitro* using the mMessage T7 kit (Ambion; AM1340) and the Poly(A) Tailing Kit (Ambion; AM1350). mRNA was purified with the MEGAclear kit (Ambion).

### *In situ* hybridization

The spatial and temporal patterns of expression of selected genes were analyzed by whole-mount *in situ* hybridization (WISH) of zebrafish embryos following standard protocols (Jacobs et al., 2011). These methods were adapted to evaluate *Hemogen* expression in tissues, peripheral blood smears and pronephric kidney prints prepared from euthanized adult fish [200 mg l^−1^ tricaine methane sulfonate (MS222; Sigma-Aldrich, 886862)] (Detrich and Yergeau, 2004; Gupta and Mullins, 2010). For sectioning, embryos and tissues were embedded in a solution containing 0.25 g gelatin, 30 g albumin, 22 g sucrose, 2.5% glutaraldehyde (v/v) per 100 ml phosphate buffered saline (PBS). Sections were cut with a vibrating blade microtome (Leica, Wetzlar, Germany; VT1000S). Digoxigenin-labeled antisense and sense RNA probes were transcribed from zebrafish cDNA clones using the DIG RNA Labeling Kit (Roche Diagnostics, Indianapolis, USA; 11175025910).

### Indirect immunofluorescence

Zebrafish embryos were fixed in 4% paraformaldehyde (PFA) at 48 hpf. Embryos were incubated with 1:1000 rabbit anti-Hemogen primary antibody (Aviva, San Diego, USA; ARP57794_P050) followed by 1:1000 goat anti-rabbit IgG Alexafluor 488 secondary antibody (Life Technologies; A11034) as previously described (Westerfield, 2000). The specificity of the Hemogen antibody was validated both by Clontech (Mountain View, USA) and by our laboratory by western blotting of zebrafish protein extracts.

### Hemoglobin staining

To detect red blood cells in circulation, embryos were stained with o-dianisidine (Iuchi and Yamamoto, 1983) or diaminofluorene (McGuckin et al., 2003).

### Western blotting

Total embryonic protein was prepared for sodium dodecyl sulfate polyacrylamide gel electrophoresis (SDS-PAGE) from dechorionated, 33 hpf embryos (*n=*80) by homogenization in lithium dodecyl sulfate (LDS) Bolt buffer (Life Technologies; B007) and NuPAGE reducing agent (Life Technologies; NP0009) using a pestle and microcentrifuge tube (USA Scientific, Ocala, USA; 1415-5390). Samples were boiled for 3 min and centrifuged at top speed in a centrifuge for 2 min. Aliquots (15 µg) were electrophoresed on a 4-12% SDS polyacrylamide gel, and the separated proteins were transferred to a polyvinylidene difluoride (PVDF) membrane with the iBlot system (Life Technologies; IB21001). Membranes were blocked in maleic acid blocking buffer (2% Roche blocking reagent, 2% BSA, 0.2% heat treated goat serum, 0.1% Tween-20) for 1 h at room temperature and then incubated overnight at 4°C with 1:1000 rabbit anti-Hemogen (Aviva; ARP57794_P050) or with 1:1000 mouse anti-GAPDH (Aviva; OAE00006) antibodies. Membranes were washed in TBST (Tris-buffered saline and Tween 20) and incubated for 2 h with horseradish peroxidase (HRP)-conjugated goat anti-rabbit IgG (H&L) (Aviva; ASP00001) or HRP-conjugated goat anti-mouse IgG (H&L) (Aviva; OARA04973), respectively. Bound antibodies were detected with the Amersham ECL Western Blotting Analysis System (GE Healthcare; RPN2106) on CL-X Posure film (Thermo Fisher Scientific; 34091).

### *Tol2* generation of *Tg(Hemgn:mCherry)* zebrafish

To identify the regulatory elements that drive *Hemogen* expression in zebrafish, three different *Tg(Hemgn:mCherry)* reporter plasmids were created using Gateway Cloning Technology (Invitrogen; 11791020) (Hartley et al., 2000). First, the proximal *Hemogen* promoter (∼2.2 kb) was amplified from wild-type SAT zebrafish using 1 µM primers (Table S1). The promoter sequence spanned the upstream, non-coding region before, but not including, the *Hemogen* translation start codon. The promoter was cloned between *KpnI/SpeI* restriction sites in the p5e-MCS vector (Tol2kit, http://tol2kit.genetics.utah.edu; #228) using the Tol2kit vector system (Kwan et al., 2007) to generate the entry clone, p5e-Hemgn-1. The resulting plasmid was digested with *NaeI/KpnI* or *NaeI/SpeI* to remove each of two conserved non-coding elements (CNE1 or CNE2) from the promoter. Each new construct was blunt-ended with Q5 Hot Start High-Fidelity 2× Master Mix (NEB) and ligated with T4 DNA Ligase (NEB) to create p5e-Hemgn-2 and p5e-Hemgn-3. Each of the three entry clones were cloned in front of the *mCherry* gene within the pDestTol2CG2 destination vector (Tol2kit; #395). The pCS2FA-transposase clone (Tol2kit; #396) was digested with *PmeI*, and *Tol2* transposase mRNA was transcribed, capped and polyadenylated *in vitro* using the mMessage SP6 kit (Ambion; AM1340) and the Poly(A) Tailing Kit (Ambion; AM1350). mRNA was purified by precipitation using 2.5 M LiCl. Transposase mRNA (37 ng µl^−1^) and each of the Tg(*Hemgn:mCherry,myl7:EGFP)* expression clones (25 ng µl^−1^) were co-injected into one-cell wild-type zebrafish embryos. Founders were raised and out-crossed to wild-type TU zebrafish for two generations.

### CRISPR/Cas9 generation of transgenic and mutant zebrafish

Optimal targets for CRISPR-Cas9 mutagenesis were identified within the first and third exons of zebrafish *Hemogen* using the program CHOPCHOP (Labun et al., 2016; Montague et al., 2014). The templates for multiple small guide RNAs were produced by a cloning-free method as previously described (Table S1) (Hruscha et al., 2013; Talbot and Amacher, 2014). Guide RNAs were transcribed with the T7 MaxiScript Kit (Ambion; AM1312) and purified by LiCl precipitation.

A donor construct for homology directed repair was created containing the *mCherry* gene and polyadenylation signal flanked by 199 bp and 253 bp homology arms that were PCR amplified from the sequence surrounding exon 1 of *Hemogen* from wild-type AB zebrafish (Table S1). The homology arms and *mCherry* gene were PCR amplified with primers that added *AvrII* and *ClaI* restriction sites, ligated and cloned into the pGem-T Easy vector (Promega). *Tg(Lcr:EGFP)^cz3325Tg^* embryos were co-injected at the single-cell stage with *EcoRI* linearized donor plasmid (25 ng µl^−1^), two exon-1 targeting guide RNAs (150 ng µl^−1^) and Cas9 mRNA (300 ng µl^−1^) (Trilink, San Diego, USA). Embryos were checked for fluorescence between 1 and 3 dpf. To confirm integration, the locus was PCR amplified with internal and external primers (Table S1) and cloned into the pGem-T Easy vector for sequencing.

Wild-type (TU) embryos were co-injected with a guide RNA (150 ng µl^−1^) targeting exon 3, Cas9 mRNA (300 ng µl^−1^) and *mCherry* mRNA (30 ng µl^−1^) to identify successful injections. Embryos were raised and adults were tail-clipped for haplotyping by high-resolution melting analysis (HRMA) as previously described (Talbot and Amacher, 2014). PCR amplification was run using 1 µM primers (Table S1) with PowerUp SYBR MasterMix (Applied Biosystems, Foster City, USA; A25742) on a QuantStudio 3 Real-time PCR system (Thermo Fisher Scientific; A28137). Founder mutants were outcrossed to TU fish. The offspring were raised and mutations were characterized by HRMA and sequencing of the locus.

### Imaging of zebrafish embryos

Fixed embryos were mounted in 80% glycerol and imaged with a dissecting microscope (Nikon; SMZ-U) and a CCD digital camera (Diagnostic Instruments, Sterling Heights, USA; SPOT32). Live embryos were embedded in 0.1% agarose in embryo medium (EB) with 0.01% tricaine and imaged with an epifluorescence-equipped microscope (Nikon; Eclipse E800). Movies (0.01 s interval) and time-lapse images (1 min interval) were obtained using a Photometrics Scientific CoolSNAP EZ camera and NIKON NIS-Elements AR 4.20 software. Methods for *in vivo* flow analyses were adapted to quantify fluorescently labeled red blood cells in MO-injected *Tg(Lcr:EGFP)^cz3325Tg^* zebrafish (Schwerte et al., 2003; Zeng et al., 2012). Briefly, 100 frame videos were taken set at a 500 μs exposure time with no delay. The field of view (20×) was centered on the dorsal aorta adjacent to the cloaca. The summed maximum intensity images of all frames were used to create ‘casts’ of the dorsal aorta and the average volume was calculated assuming cylindrical vasculature. EGFP+ cells were converted to binary objects (6.66 µm diameter, contrast 180) and counted within the region of interest.

### qRT-PCR

RNA was purified from adult zebrafish tissues or 10-30 pooled embryos at 3 or 4 dpf in TriZol (Sigma-Aldrich; T9424) using the PureLink RNA purification Kit (Ambion). DNase treated RNA was reverse transcribed with a polyT_(23)_ primer using Protoscript II RT-PCR kit (NEB; M0368S). Target genes were amplified in triplicate from cDNA by qRT-PCR with 1 µM primers (Table S1). Standard curves were generated to confirm primer efficiencies. Target gene expression was normalized to *beta-actin* for comparison by the ΔΔCt method. Three or four biological replicates were used for each treatment for statistical comparisons.

### Statistical analyses

Data were analyzed as means±s.e.m. or means±s.d. as noted. Statistical tests applied to the results are provided with each experiment. Differences with a *P-*value≤0.05 were considered significant.

### GenBank accession numbers

Zebrafish *Hemgn* isoform 1, JZ970258; zebrafish *Hemgn* isoform 2, JZ970260; zebrafish *Hemgn* isoform 3, JZ970259; and zebrafish *Hemgn* isoform 4, JZ970257.

### Zebrafish ZFIN IDs

Transgenic construct *Tg(hemgn:mCherry,myl7:EGFP)*, ZDB-TGCONSTRCT-170726-1; zebrafish line *nuz1Tg*, ZDB-ALT-170726-1; zebrafish line *hemgn^nuz2^*, ZDB-ALT-170726-2; zebrafish line *hemgn^nuz3^*, ZDB-ALT-170726-3; zebrafish line *hemgn^nuz4^*, ZDB-ALT-170726-4. All transgenic lines, with the exception of *nuz3*, are available through the Zebrafish International Resource Center.

## Supplementary Material

Supplementary information
