# Supplementary material for: Divergent Hemogen genes of teleosts and mammals share conserved roles in erythropoiesis: analysis using transgenic and mutant zebrafish
Source: Biol Open. 2018 Aug 10;7(8):bio035576. doi: 10.1242/bio.035576 (PMC6124579; doi:10.1242/bio.035576)
Supplement: Supplementary information [file biolopen-7-035576-s1.pdf]

```
H. sapiens    1   M-D-LGKDQSHLKHHQTPDPHQEENHSPEVIG-TWSLRNRELLRKRKAEVHEKETS-QWLFG-EQKK---RKQQR-TGKG
M. musculus   1   M-D-MGKGRPRLKLPQMPEAHPQKSCAPDIIG-SWSLRNREQLRKRKAEAQGRQTS-QWLLG-EQKK---RKYQR-TGKG
G. aculateus  1   MEETLQQEK--------QNSDYENSNEDQG-GIRRRLRDRDLLKKRKAEAEEKETN-QWVWG--SQR---KRPRAESGTK
G. gallus     1   MDSLGRDH--DYSNTSLPSSTAHEENAEPGSTMLHRLRDRELLRKRKAEAEEKD-SIQWALG-EHER---SKRQR-RGQG
C. milii      1   ME--------DIDN-AMEKLTQKSSSKETPDISPRRLRDREKLKKRKLEMQVKEPS-QWVTV----VESKKKR-------
D. rerio WT   1   MEDPLEKEI----------PPTEIKDSDEG-GIRRRLRDRDLLKKRKAEAEEKATN-QWVYGAQSIKRVKKRTT--TGTP
                                                      Coiled-Coil     Spliced         NLS
H. sapiens    72  NRRGRKR-----------QQNTELKVEPQPQIEKEIVEKALAPIEKKTEPPGSITKVFPSVASPQKVVPEEHFSEICQES
M. musculus   72  NKRGRKR-----------QGNVEQKAEPWSQTERERVQE-VLVSAEEETEHPGNSATEALPLVPSP----TKAVPADQCS
G. aculateus  66  -RRGRPKKSEPAPEICVN----QEE-AAVPQEAPA-A----VVPEPAEVVPYQTPESLSPV---LDGESRPSSVPAVP-A
G. gallus     73  ARRGKGRE---------------------------------HVVEPAP--------------------------------
C. milii      60  GRRGKSHSKK----------------------------------------------------------------------
D. rerio WT   67  GRKGRPRK-EPIVIAEDL-GLAQEVDPSPITIPVTEP----ELPTIAEIEPLQ-P-------------------------

H. sapiens    141 NIYQENFSEYQEIAVQNHSSETCQHVSEPEDLSPKMYQEISVLQDNSSKICQDMKEPEDNSPNTCQ-VISVI-QDHPFK-
M. musculus   136 EA-HQESIQCQERAIQNHSQTHLSPTTCQGIAVLQHSPKMCQ----------DMAEPEVFSPNMCQ-ETAVP-QTYPPKA
G. aculateus  131 A--PAPRPLL--GSIQSPVFAASPTPPAPVLPTPDLFSPSAPVSFPVKLA--DFAPVSVPYSAPDAVPVLT---------
G. gallus     88  --------------------------------------------------------------------------------
C. milii      69  --------------------------------------------------------------------------------
D. rerio WT   115 --------------------------------------------------------------------------------

H. sapiens    219 MYQDMAKREDLAPKMCQEAAVPKILPCPTSEDTADLAGCSLQAYPKPDVPKGYILDTDQNPAEPEEYNETDQGIAETEG-
M. musculus   203 L-EEMAAAEPLSPKMCQETTVSPNHSSKVPQDMAGPEALSPNMCQEPTVPQEHTLKMCHDVARPEVLSPKTHQEMAVPKA
G. aculateus  196 ------QAPVPTAAPP----------------------------------------------------------------
G. gallus     88  --------------------------------------------------------------------------------
C. milii      69  --------------------------------------------------------------------------------
D. rerio WT   115 --------------------------------------------------------------------------------

H. sapiens    298 LFPKI------QEIAEPKDLSTKTHQESAEPKYLPHKTCN----------------------EIIVPKAPSHKTIQETPH
M. musculus   282 FPCVTPGDAAGLEGCAPKALPQSDVAEGCPLDTTPTSVTPEQTTSDPDLGMAVTEGFFSEARECTVSEGVSTKTHQEAVE
G. aculateus  206 -----------------------------------------------------------------------------QED
G. gallus     88  --------------------------------------------------------------------------------
C. milii      69  --------------------------------------------------------------------------------
D. rerio WT   115 --------------------------------------------------------------------------------

H. sapiens    349 SEDY------SIEINQETPGSEK-YSPETYQEIPGLEEYSPEIYQETSQLEEYSPEIYQETPGPEDLSTETYKNKDVPKE
M. musculus   362 PEFISHETYKEFTVPIV--SSQK-----TIQESPEPEQYSPETCQPIPGPENYSLETCHEMSGPEDLSIKTCQDREEPKH
G. aculateus  209 TLFT-------------ESQSRKVLD------------------------------------------------------
G. gallus     88  --------------------QSKPQPNP-QPPKKEEAEKMSSAAMQQAESSQ----------------------------
C. milii      69  ---------------------AESEPEIVQQLE-DEAEDRPLIISESERPECAEESIKLSVIEEAPTTSADLTKEIKSPN
D. rerio WT   117 -------------------------VEPQQELVPEEAISEKPQE------------------------------------

H. sapiens    422 CFPEPHQETGGPQGQDPKAHQEDAKDAYTFP-QEMKEKPK--EEPGIPAIL---NE-------SHPENDVY-SYVLF 484
M. musculus   435 SLPEGAQKVGGAQGQDADAQDSENAGAFSQDFTEMEEENKADQDPEAPASPQGSQE-------TCPENGIY-SSALF 503
G. aculateus  229 --------QVLIKDLGPGDEEDIYSSNDQTANEDLIGTSAI--N-VPEQNK----MFSIPTLSPTPPPQKYFPGNLF 290
G. gallus     119 -----MDVQDLFAGVQLSDLEGIL--GSQ-SP--LGEED----ML-KLADEIQEV-LNTSLEKNEPDNDVY-SSSLF 180
C. milii      128 --------TEFLENLKFQL-EDLP------------EENMPLEE-------------------------KH-PVYL  156
D. rerio WT   140 --------EFLIEDLGPDEEEDMP----QKHLVIDTGDDEKPYNDVPEQSSIAIPMFAPAPDSSQPDNLSLTENLLF 198
D. rerio Δ12 140 --------EFLIEDLGPD----MP----QKHLVIDTGDDEKPYNDVPEQSSIAIPMFAPAPDSSQPDNLSLTENLLF 194
D. rerio Δ5  140 --------EFLIEDLGPGGRYASKTPGYRYRRRREAI                                         168
                                      Acidic Domain
```

Figure S1. Alignment of the amino acid sequences of wild-type and mutant Hemogens in zebrafish with the orthologous proteins from other vertebrate species. Transactivation domain (TAD) motifs are boxed and were identified in human and zebrafish Hemogens by ϕϕxxϕ or ϕxxϕϕ, where ϕ is a bulky hydrophobic motif. Alleles are shown for *Hemgn*^{nuz2} (Δ5) and *Hemgn*^{nuz4} (Δ12) mutant zebrafish lines. Predicted motifs: green, coiled coil; blue, nuclear localization signal; maroon, four residues introduced by alternative splicing; yellow, tandem peptide repeats; box, TAD motif; purple, TAD motif conserved in teleosts; bold italic, acidic region; red, frameshifted residues; red dashes, deletion. Species abbreviations: *H. sapiens, Homo sapiens; M. musculus, Mus musculus; G. aculateus, Gasterosteus aculeatus; G. gallus, Gallus gallus; C. milii, Callorhinchus milii; D. rerio, Danio rerio*

A

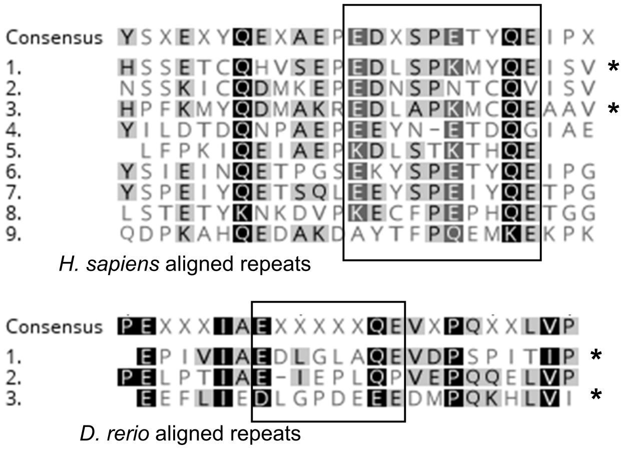

B

Amino Acid Composition Zebrafish						
	Total Protein		Repeats		Acidic Region	
Ala (A)	N =11	5.6%	N = 4	4.8%	N = 1	2.1%
Arg (R)	N =11	5.6%	N = 0	0.0%	N = 0	0.0%
Asn (N)	N = 4	2.0%	N = 0	0.0%	N = 1	2.1%
Asp (D)	N =16	8.1%	N = 6	7.2%	N = 7	14.9%
Cys (C)	N = 0	0.0%	N = 0	0.0%	N = 0	0.0%
Gln (Q)	N =10	5.1%	N = 6	7.2%	N = 2	4.3%
Glu (E)	N =29	14.6%	N =17	20.5%	N = 11	23.4%
Gly (G)	N = 9	4.5%	N = 3	3.6%	N = 2	4.3%
His (H)	N = 1	0.5%	N = 1	1.2%	N = 1	2.1%
Ile (I)	N =15	7.6%	N = 9	10.8%	N = 3	6.4%
Leu (L)	N =16	8.1%	N = 8	9.6%	N = 3	6.4%
Lys (K)	N =14	7.1%	N = 2	2.4%	N = 3	6.4%
Met (M)	N = 3	1.5%	N = 1	1.2%	N = 1	2.1%
Phe (F)	N = 3	1.5%	N = 1	1.2%	N = 1	2.1%
Pro (P)	N =24	12.1%	N =13	15.7%	N = 6	12.8%
Ser (S)	N = 9	4.5%	N = 2	2.4%	N = 1	2.1%
Thr (T)	N =11	5.6%	N = 4	4.8%	N = 1	2.1%
Trp (W)	N = 1	0.5%	N = 0	0.0%	N = 0	0.0%
Tyr (Y)	N = 2	1.0%	N = 0	0.0%	N = 1	2.1%
Val (V)	N = 9	4.5%	N = 6	7.2%	N = 2	4.3%

Figure S2. Analysis of peptide repeats (A) Alignment of predicted tandem peptide repeats from zebrafish and human Hemogens. Conserved residues are shaded black. Each predicted repeat in human Hemogen can be divided into two more repeats. Conserved regions of peptide repeats between zebrafish and human Hemogens are boxed. Repeats are most similar within species but repeats 1 and 3 are similar between human and zebrafish Hemogens (marked with asterisks). (B) Amino acid composition of zebrafish Hemogen. The repeat region is enriched for glutamic acid and proline. The acidic C-terminal repeat is enriched for glutamic acid and aspartic acid.

Biology Open • Supplementary information

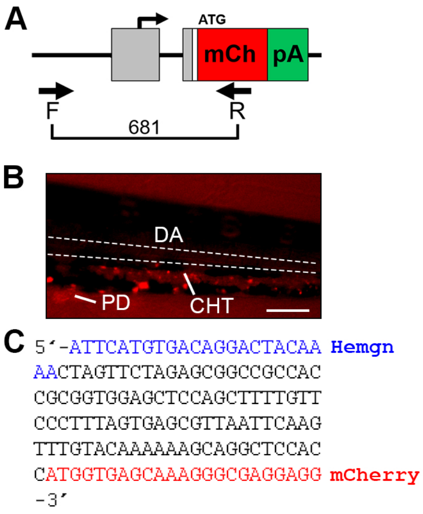

Figure S3. CRISPR/Cas9-mediated replacement of zebrafish *Hemogen* with the *mCherry* transgene recapitulates endogenous *Hemogen* expression in zebrafish. (A) Schematic showing insertion of the *mCherry* transgene at the CRISPR target site within exon 1 of zebrafish *Hemogen*. Integration of the *mCherry* transgene was confirmed by sequencing the locus with internal and external primers (arrows; Table S1). (B) 3 dpf. Representative image of the tail segment. mCherry+ mutant cells were present in the CHT and the pronephric duct (PD) and at a low frequency in circulation in the dorsal aorta (DA, dashed outline) (n = 15 embryos). (C) Sequence across the insertion, showing part of the *Hemogen* promoter (blue), the first 7 codons of the mCherry transgene (red), and a linker sequence (black). Scale Bar = 100 µm (B).

Biology Open • Supplementary information

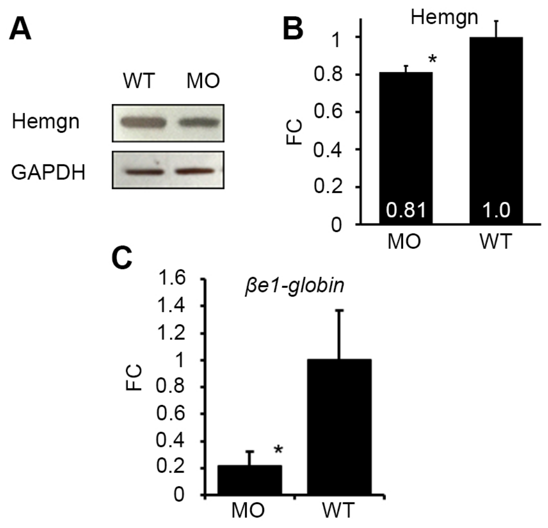

Figure S4. (A) Representative Western blot of Hemogen from pooled morphants (MO) or wild-type (WT) embryos at 33 hpf. (B) Average Hemogen protein expression from three experiments. GAPDH served as the internal control. (*, $P \leq 0.05$, Student's *t* test). (C) Relative *βe1-globin* expression in pooled morphant or wild-type embryos at 3 dpf as determined by qRT-PCR. Four samples of 10 pooled embryos were amplified per treatment. Signals were normalized to *β-actin* and shown relative to wild-type. Error bars represent the standard deviation (*, $P \leq 0.05$, Student's *t* test).

Biology Open • Supplementary information

A Genotypic ratios

	Δ5		Δ12	
+/-	30	54.5%	19	59.4%
-/-	12	21.8%	5	15.6%
+/+	13	23.6%	8	25%
Total	55		32	

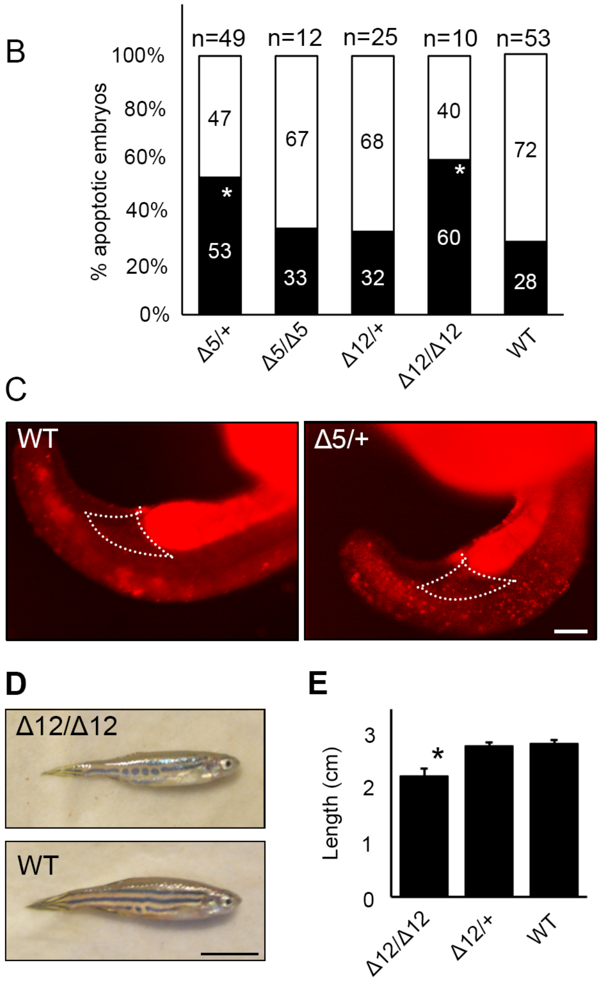

Biology Open (2018): doi:10.1242/bio.035576: Supplementary information

Supplemental Figure 5. *Hemogen* mutant zebrafish have increased cell death during embryonic development. (A) Genotypic ratios of 2 dpf embryos produced from heterozygous incrosses of *Hemgn*^{nuz3} ($\Delta 5$) or *Hemgn*^{nuz4} ($\Delta 12$) mutants. (B) Proportion of genotyped mutants and wild-type sibling embryos at 2 dpf that were apoptotic (black) or phenotypically normal (white) (* $P \leq 0.05$, ** $P \leq 0.005$, Chi square). (~~B~~C) Acridine orange staining for apoptotic cells is increased in the bodies and in the peripheral blood island (outlined) in 20 hpf heterozygous *Hemgn*^{nuz2} mutant zebrafish (n = 3) compared to wild-type siblings (n = 3). (D) Comparison of adult wild-type and homozygous *Hemgn*^{nuz4} ($\Delta 12$) mutant. (E) Average body length of adult wild-type and *Hemgn*^{nuz4} ($\Delta 12$) mutants. Error bars represent standard error (*, $P \leq 0.05$, Student's *t* test). Scale bars = 100 µm (E), 50 mm (D)

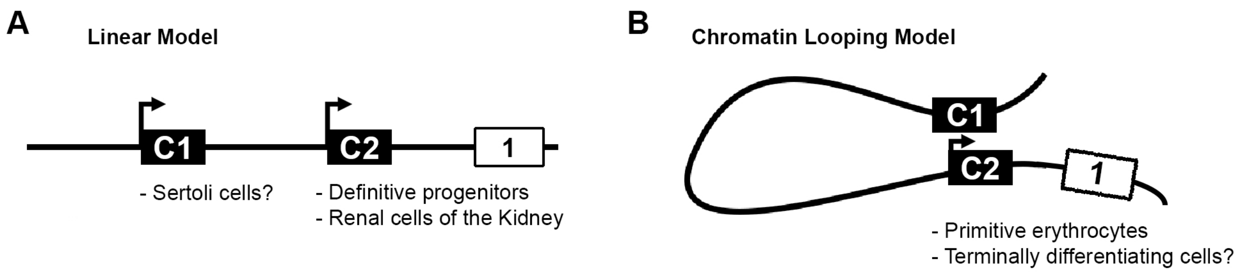

Supplemental Figure 6. Proposed models for regulation of *Hemogen* expression by promoter elements. (A) Linear, two-promoter model. (B) Chromatin looping model.

Biology Open • Supplementary information

Table S1. Sequences of primer and guide oligonucleotides used in experiments

Gene	Oligo Sequence (5' – 3')	Method
Hemgn F1<br>*Hemgn F2*<br>*Hemgn F3*<br>*Hemgn R1*<br>*Hemgn R2*	CTTTCTTCTGTGAGTATTGTGC<br>GAGAAAGAGATCCCACCAACTG<br>GACATGATTGTGAACACGCCC<br>TTGTTTCCATAGTAAGGAGGTG<br>TCTGAGTCGCCGCCGAATTCC	RT-PCR of *Hemgn*
Hemgn F1<br>*Hemgn F2*<br>*Hemgn R*	GACATGATTGTGAACACGCCC<br>CTGTGAGTATTGTGCCAAGTCC<br>TCTGAGTCGCCGCCGAATTCC	qRT-PCR of *Hemgn*
Hemgn-Kpn1F<br>*Hemgn-Spe1-R*	ATCATGGGTACCCACATCCAGAAATGAGACAT<br>ATCATGACTAGTTTTGTAGTCCTGTCACATGA	PCR Promoter
Hemgn F<br>*mCherry R*	CTGTGAGTATTGTGCCAAGTCC<br>GAACTCCTTGATGATGGCC	RT-PCR transgene
HemgnMM F4<br>*Hemgn R1*	ACCATGGAGGATCCGCTGGAGAAAGAGA<br>TTGTTTCCATAGTAAGGAGGTG	zHemgn rescue cDNA
βe1-globin F<br>*βe1-globin R*<br>*β-actin F*<br>*β -actin R*	TCGCCAAGGCTGACTACGA<br>CGGCATTGTAGGTTTCCAA<br>CGAGCAGGAGATGGGAACC<br>CAACGGAAACGCTCATTGC	qRT-PCR of Morphants
LarmF<br>*LarmR-AvrII-R*<br>*Rarm-ClaI-F*<br>*RarmR*<br>*mCherry-AvrII-F*<br>*PolyA-ClaI-R*	CTGTGAGTATTGTGCCAAGTCC<br>ATCATGCCTAGGGTCTTCCATTTTGTAGTCC<br>ATGTACATCGATCCTTAGCATTAAACATCAATCAC<br>CCATGCCTAGTGTCAGGATC<br>ATCATGCCTAGGATGGTGAGCAAGGGCG<br>ATGTACATCGATCTTGTTTATTGCAGCTTATAATGGTTAC	Constructing Donor Plasmid
Hemgn F<br>*mCherry R*	GCTCGCTTGTTGTTTACTCT<br>GAACTCCTTGATGATGGCC	PCR Knock-in
sgRNA	AAAAGCACCGACTCGGTGCCACTTTTTCAAGTTGATAACG<br>GACTAGCCTTATTTTAACTTGCTATTTCTAGCTCTAAAAC	CRISPR template
sgRNA Hemgn ex1a	GAAATTAATACGACTCACTATAGGTGGGATCTCTTTCTCC<br>AAGTTTTAGAGCTAGAAATAGC	CRISPR template
sgRNA Hemgn ex1b	GAAATTAATACGACTCACTATAGGAATAAAAGATTCAGAT<br>GAGTTTTAGAGCTAGAAATAGC	CRISPR template
sgRNA Hemgn ex3	GAAATTAATACGACTCACTATAGGATCTGGGGCCAGATG<br>AGGGTTTTAGAGCTAGAAATAGC	CRISPR template
Hemgn ex3 F<br>*Hemgn ex3 R*	GGTGCCTGAAGAAGCAATAAGTG<br>CATTCATGAACAAGACGTTTCAGC	HRMA
Hemgn ex1 F<br>*Hemgn ex1 R*	GCATGAATGTAAGCGGGC<br>GTGATTGATGTTTAATGCTAAGG	HRMA
Hemgn WT F<br>*Hemgn Δ5 F*<br>*Hemgn Δ12 F*<br>*Hemgn Both F*<br>*Hemgn Both R*	TGAGGATCTGGGGCCAGATG<br>GGATCTGGGGCCAGGAG<br>AGGATCTGGGGCCAGATATGC<br>GATTGAGGATCTGGGGCCAG<br>GGTGCTGGAGCAAACATTGG	qRT-PCR of mutant alleles

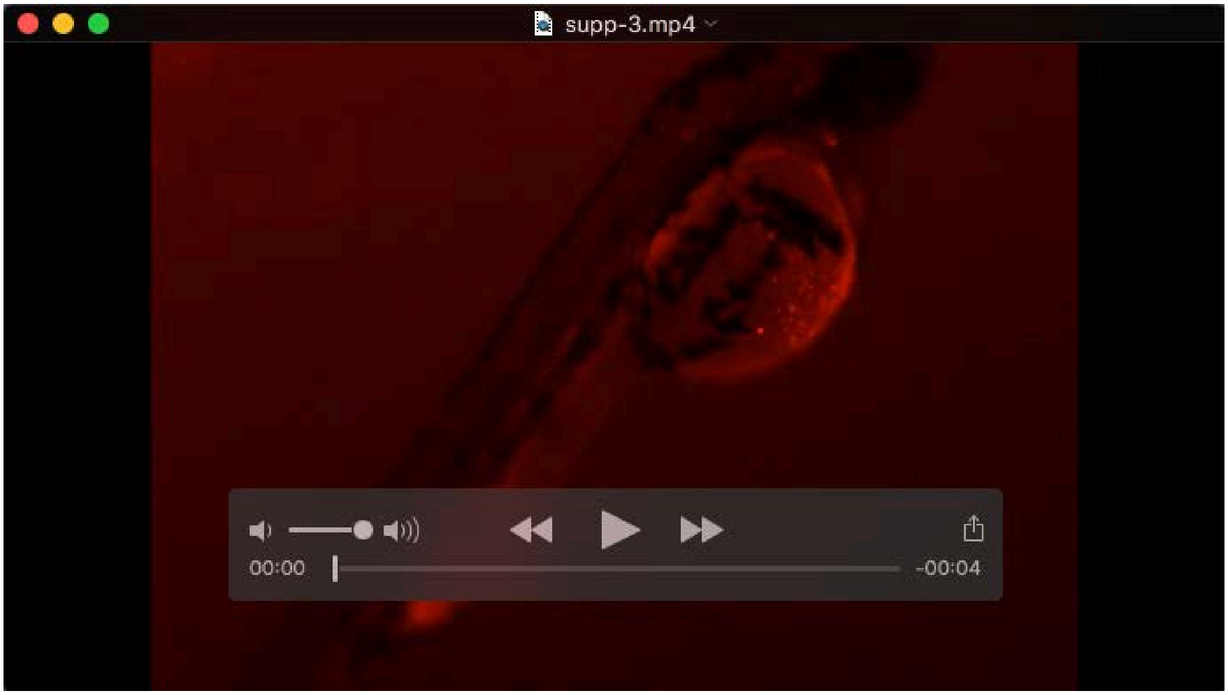

Movie S1. Circulating erythrocytes in *Tol2*-generated transgenic *Tg(Hemgn-1:mCherry,myl7:EGFP)* zebrafish at 2 dpf. 4x magnification.

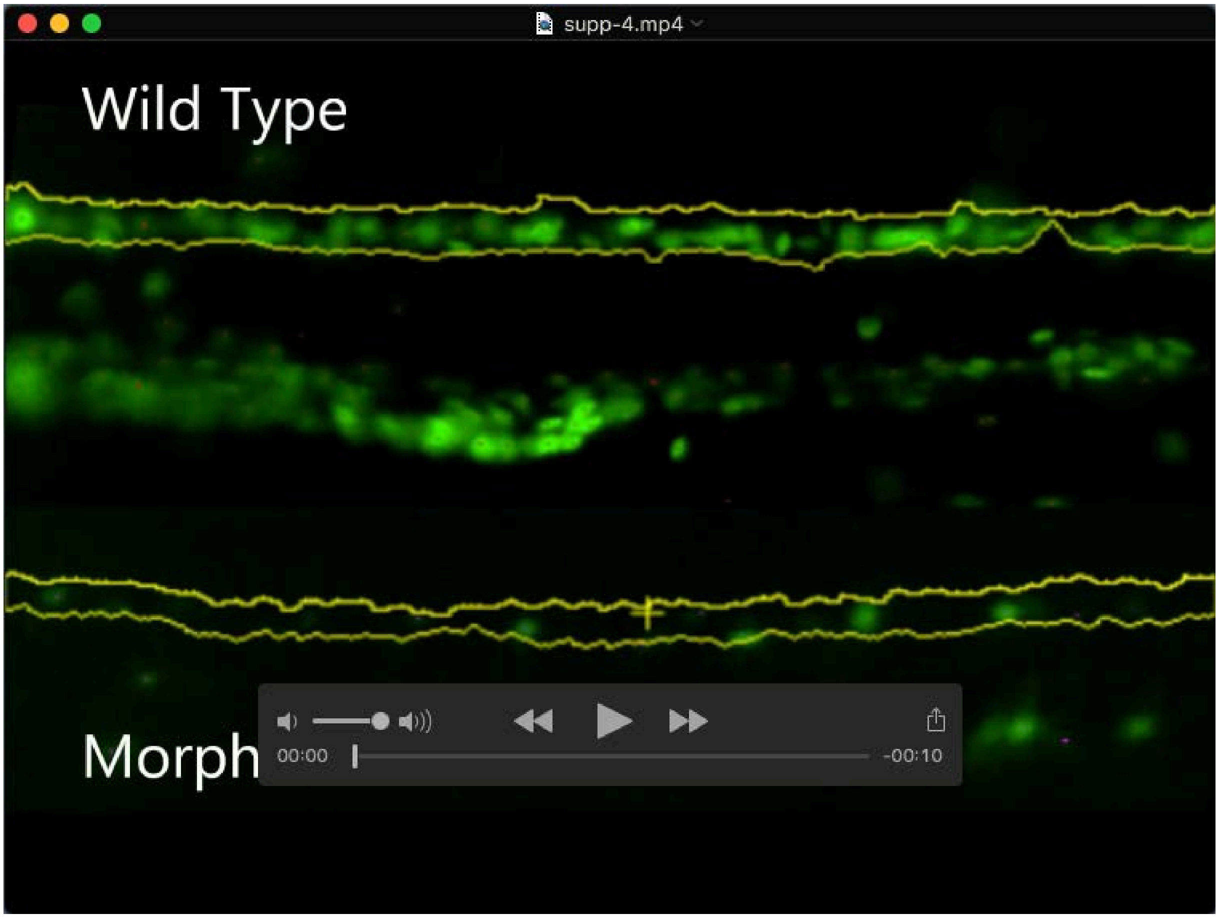

Movie S2. Comparison of circulating EGFP+ erythrocytes in the dorsal aorta of *Hemogen* morphant and wild-type *Tg(Lcr:EGFP)*^{cz3325Tg} zebrafish embryos at 3 dpf. The dorsal aorta is highlighted, and EGFP+ erythrocytes are marked with a dot. 20x magnification.

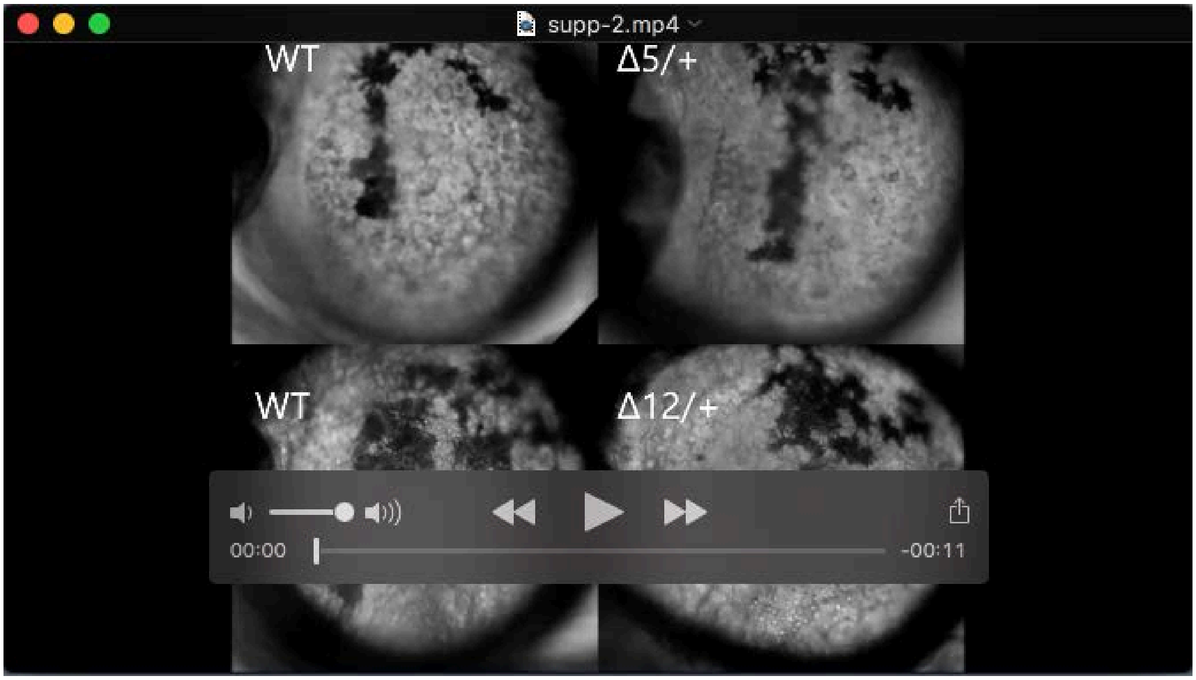

Movie S3. Comparison of circulating erythrocytes in, *Hemgn*$^{nuz2/+}$ zebrafish embryos (*Δ5* frameshift), *Hemgn*$^{nuz4/+}$ embryos (*Δ12* deletion) and wild-type siblings at 24 hpf. 10x magnification.
